# IRE1α RNase activity is critical for early embryo development by degrading maternal transcripts

**DOI:** 10.1093/nar/gkaf520

**Published:** 2025-06-18

**Authors:** Chao Li, Yong-Peng Tan, Di Gao, Ruibao Su, Ke Xu, Shu-Chen Liu, Xu-Feng Li, You-Hui Lu, Li-Tao Yi, Guang Wang, Xiang-Hong Ou, Tie-Gang Meng, Qing-Yuan Sun

**Affiliations:** Guangdong Second Provincial General Hospital, Postdoctoral Research Station of Basic Medicine, School of Medicine, Jinan University, Guangzhou 510317, China; Guangzhou Key Laboratory of Metabolic Diseases and Reproductive Health, Guangdong–Hong Kong Metabolism & Reproduction Joint Laboratory, Reproductive Medicine Center, Guangdong Second Provincial General Hospital, Guangzhou 510317, China; Guangzhou Key Laboratory of Metabolic Diseases and Reproductive Health, Guangdong–Hong Kong Metabolism & Reproduction Joint Laboratory, Reproductive Medicine Center, Guangdong Second Provincial General Hospital, Guangzhou 510317, China; Guangzhou Key Laboratory of Metabolic Diseases and Reproductive Health, Guangdong–Hong Kong Metabolism & Reproduction Joint Laboratory, Reproductive Medicine Center, Guangdong Second Provincial General Hospital, Guangzhou 510317, China; Guangzhou Key Laboratory of Metabolic Diseases and Reproductive Health, Guangdong–Hong Kong Metabolism & Reproduction Joint Laboratory, Reproductive Medicine Center, Guangdong Second Provincial General Hospital, Guangzhou 510317, China; International Joint Laboratory for Embryonic Development & Prenatal Medicine, Division of Histology and Embryology, Medical College, Jinan University, Guangzhou 510632, China; Guangzhou Key Laboratory of Metabolic Diseases and Reproductive Health, Guangdong–Hong Kong Metabolism & Reproduction Joint Laboratory, Reproductive Medicine Center, Guangdong Second Provincial General Hospital, Guangzhou 510317, China; Department of Developmental Biology, School of Basic Medical Sciences, Southern Medical University, Guangzhou 510515, China; Guangzhou Key Laboratory of Metabolic Diseases and Reproductive Health, Guangdong–Hong Kong Metabolism & Reproduction Joint Laboratory, Reproductive Medicine Center, Guangdong Second Provincial General Hospital, Guangzhou 510317, China; Guangzhou Key Laboratory of Metabolic Diseases and Reproductive Health, Guangdong–Hong Kong Metabolism & Reproduction Joint Laboratory, Reproductive Medicine Center, Guangdong Second Provincial General Hospital, Guangzhou 510317, China; Department of Developmental Biology, School of Basic Medical Sciences, Southern Medical University, Guangzhou 510515, China; Guangzhou Key Laboratory of Metabolic Diseases and Reproductive Health, Guangdong–Hong Kong Metabolism & Reproduction Joint Laboratory, Reproductive Medicine Center, Guangdong Second Provincial General Hospital, Guangzhou 510317, China; International Joint Laboratory for Embryonic Development & Prenatal Medicine, Division of Histology and Embryology, Medical College, Jinan University, Guangzhou 510632, China; Guangzhou Key Laboratory of Metabolic Diseases and Reproductive Health, Guangdong–Hong Kong Metabolism & Reproduction Joint Laboratory, Reproductive Medicine Center, Guangdong Second Provincial General Hospital, Guangzhou 510317, China; International Joint Laboratory for Embryonic Development & Prenatal Medicine, Division of Histology and Embryology, Medical College, Jinan University, Guangzhou 510632, China; Guangzhou Key Laboratory of Metabolic Diseases and Reproductive Health, Guangdong–Hong Kong Metabolism & Reproduction Joint Laboratory, Reproductive Medicine Center, Guangdong Second Provincial General Hospital, Guangzhou 510317, China; International Joint Laboratory for Embryonic Development & Prenatal Medicine, Division of Histology and Embryology, Medical College, Jinan University, Guangzhou 510632, China; Guangzhou Key Laboratory of Metabolic Diseases and Reproductive Health, Guangdong–Hong Kong Metabolism & Reproduction Joint Laboratory, Reproductive Medicine Center, Guangdong Second Provincial General Hospital, Guangzhou 510317, China; International Joint Laboratory for Embryonic Development & Prenatal Medicine, Division of Histology and Embryology, Medical College, Jinan University, Guangzhou 510632, China; Department of Developmental Biology, School of Basic Medical Sciences, Southern Medical University, Guangzhou 510515, China

## Abstract

During maternal-to-zygotic transition, oocytes and embryos undergo massive maternal mRNA degradation. Three key events are related to RNA degradation: oocyte meiotic resumption, fertilization, and zygotic genome activation (ZGA). In this study, we unexpectedly discover that the UPR (unfolded protein response) protein IRE1α is critical for post-fertilization maternal messenger mRNA (mRNA) degradation. IRE1α is specifically expressed from the metaphase II oocytes to four-cell embryos, with its translation dependent on the ERK1/2 pathway. Oocyte-specific deletion of the IRE1α RNase domain resulted in female infertility, characterized by embryonic developmental arrest at the one-cell or two-cell stage, and failure to degrade maternal mRNAs destined for elimination. Using IRE1α-Flag knock-in mouse model and LACE-seq, as well as *in vitro* analysis, we show that IRE1α can directly bind and cleave maternal mRNAs after fertilization. Moreover, IRE1α-mediated mRNA decay is essential for ZGA and histone modifications. This study unveils an important function of IRE1α in early embryonic development through regulated IRE1α-dependent decay, independent of the canonical IRE1α–XBP1 signaling pathway, thereby revealing a novel molecular mechanism underlying maternal mRNA degradation triggered by fertilization.

## Graphical Abstract

**Graphical Abstract:**
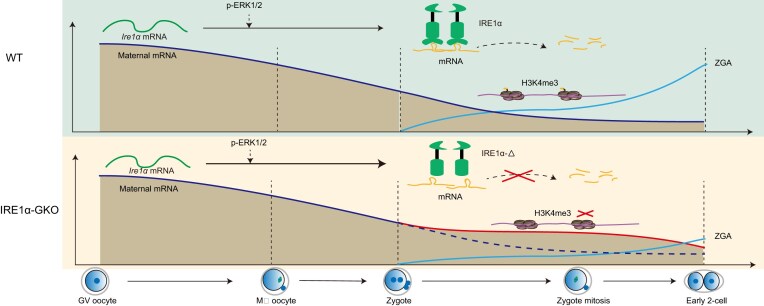

## Introduction

In all organisms, oocytes accumulate substantial RNA and protein reserves to support embryonic development after fertilization. The developmental control gradually shifts from maternal factors to zygotic gene products—a process termed maternal-to-zygotic transition (MZT) [1]. This complex process primarily involves maternal messenger RNA (mRNA) degradation and zygotic genome activation (ZGA). In different species, maternal mRNA clearance is critical for embryonic development, and failure of this process can lead to developmental arrest [2–5]. The degradation of maternal mRNA is regulated by various factors such as RNA-binding proteins, small non-coding RNAs, and RNA modifications [1]. In mice, maternal mRNA degradation occurs in three distinct waves: the first triggered by oocyte meiotic resumption, the second following fertilization, and the third initiated by zygotic factors after major ZGA [1, 4, 6]. Previous research has identified several key players in these processes, such as the CCR4–NOT deadenylase complex [7], which collaborates with oocyte-specific factors like PABPN1L and BTG4 to mediate RNA degradation [8–10]. In addition, the zygotic factor PABPN1 is involved in maternal mRNA degradation during MZT [11]. Despite these insights, the molecular mechanisms governing post-fertilization maternal mRNA elimination remain largely unexplored.

IRE1α, a transmembrane protein located on the endoplasmic reticulum (ER) membrane, primarily regulates the unfolded protein response (UPR) through its RNase domain [12]. This domain performs two critical functions: first, cleaving *Xbp1* mRNA and subsequent ligation of *Xbp1* fragments by the tRNA ligase RTCB5 [13–15], which results in the translation of an active transcription factor [16], and second, implementing regulated IRE1α-dependent decay (RIDD), which selectively reduces cellular RNA levels [17]. While XBP1s (XBP1 spliced) is believed to restore ER homeostasis [18], RIDD displays constitutive activity under basal conditions and increases with stress intensity or duration [19], and the broader functional significance of RIDD remains incompletely understood.

IRE1α is well established to function in maintaining ER homeostasis; it is also an RNA-binding protein [20], which has been shown to interact with and cleave various RNA types in diverse cellular contexts [21–24]. While its critical role in embryonic survival is evident from studies demonstrating lethality in IRE1α knockout mouse embryos by day 12.5 due to placental dysfunction [25], its specific functions during oocyte maturation and early embryonic development remain poorly understood. Notably, the unique regulatory landscape of oocytes and early embryos, characterized by dynamic RNA metabolism and translational control, suggests that IRE1α may play distinct roles in these contexts. However, whether IRE1α exhibits novel RNA targets, regulatory mechanisms, or functional outcomes during these developmental stages has not been investigated. Our study aims to address this gap by exploring the unique contributions of IRE1α to oocyte maturation and early embryogenesis, thereby uncovering previously unrecognized roles of this protein in reproductive biology.

## Materials and methods

### Mice

Care and handling of all mice were conducted in accordance with policies promulgated by the Animal Ethics Committee of Guangdong Second Provincial General Hospital. *Ire1α*^fl/fl^ mice were purchased from Riken (RBRC 05515). *Ire1α*^fl/fl^;Gcre mice were generated by crossing *Ire1α*^fl/fl^ mice with *Gdf9*-cre mice. To obtain *Ire1α*-flag knock-in mice, a 1× flag tag was inserted before the stop codon of *Ire1α* gene. This was achieved by co-injecting sgRNA1, sgRNA2, a single-stranded DNA donor, and Cas9 mRNA into zygotes. Subsequently, 15–20 zygotes were transplanted into the oviduct of pseudopregnant ICR females and kept in an specific pathogen free (SPF) environment. To confirm the successful generation of *Ire1α*-Flag mice, DNA from offsprings were amplified using primers (*Ire1α*-flag-F and *Ire1α*-flag-R) and sequenced for validation. The genotyping primers and related information are listed in Supplementary Tables S1 and S2.

### Antibodies

IRE1α antibody (CST, 14C10 for IF; Abclonal, A21021 for Wb); H3K4me3 antibody (CST, 9751); XBP1s antibody (CST, 40435); polII Ser2p antibody (Abcam, ab193468); p-eIF2α antibody (Abclonal, AP0692); HSPA5 antibody (Abclonal, A23453); 5mc antibody (Epigentek, A-1014-010); 5hmc antibody (active motif, 39792); β-actin antibody (Santa Cruz, sc-47778;); Flag antibody (Sigma, F1904); β-tubulin antibody (Abcam, ab6046); PDI antibody (Proteintech, 66422-1-Ig); and α-tubulin antibody (Sigma, F2168).

Secondary antibodies: Alexa Fluor 488-conjugated anti-mouse (Thermo Scientific, A11029) and Alexa Fluor 594-conjugated anti-rabbit (Thermo Scientific, A11012).

### Fertility test

*Ire1α* ^fl/fl^;Gcre and *Ire1α*^fl/fl^ females at 8 weeks were mated with 8-week-old wild-type (WT) males. Each cage housed one male and two females (one *Ire1α*^fl/fl^;Gcre and one *Ire1α*^fl/fl^ female). A total of three cages were monitored for fertility test for 6 months. Daily observations were conducted, and the number of litters produced by each mouse was recorded in real time.

### Oocyte/zygote collection and inhibitor treatment

The germinal vesicle (GV) oocytes were collected from ovaries using M2 medium containing 2.5 μM milrinone (MCE, HY-14252), and then washed five times with milrinone-free M2 medium and cultured in M2 medium at 37°C in a 5% CO_2_ incubator. Oocytes were treated with one of the following inhibitors: 20 μM U0126 (MCE, HY-12031A), 20 μM CHX (MCE, HY-12320), or 10 μM MG132 (MCE, HY-13259) at different developmental stages from GV to metaphase II (MII) phase, and then collected for western blotting.

For zygote collection, superovulated females were mated with fertile males, and pronuclear-stage embryos were harvested 18–20 h post-hCG injection. The embryos were cultured in KSOM medium containing either 0.1% dimethyl sulfoxide (DMSO) or one of the following inhibitors: 10 μM 4μ8c (MCE, HY-19707), 10 μM APY29 (MCE, HY-17537), 10 μM BI09 (MCE, HY-107400), and 60 μM DRB (Sigma, D1916). These embryos were collected after 10 h (PN5 zygotes) or hCG 48 h (late two-cell stage) for reverse transcriptase polymerase chain reaction (RT-PCR) or RNA-seq analysis.

### Quantitative real-time RT-PCR

Approximately 20 zygotes collected from female mice with vaginal plugs at hCG 18 h, washed three times with RNase-free phosphate buffered saline (PBS), and placed in 200-μl centrifuge tubes with minimal residual liquid, and the samples were lysed at 72°C for 3 min. The lysates were immediately cooled on ice for 1 min. RNA was reverse-transcribed into complementary DNA (cDNA) using a cDNA reverse transcription kit (Thermo, K1682). The resulting cDNA was directly used for quantitative real-time PCR (Vazyme, Q111-02). The primer sequences are listed in Supplementary Table S3.

### Construction of vector

To construct the IRE1α-FL vector, an mCherry fluorescent protein was added to the C-terminus of IRE1α to enable visualization of its expression after injection. To ensure mCherry did not interfere with IRE1α function, a P2A linker sequence was inserted between the IRE1α and mCherry sequences. The *Ire1α* sequence was amplified by PCR using cDNA from mouse oocytes as a template, while the mCherry sequence was amplified from a plasmid containing mCherry. The linearized empty plasmid, *Ire1α*, and mCherry fragments were mixed according to DNA recombination kit and transformed into DH5α competent *Escherichia coli*. The *Ire1α*-GFP vector was constructed in the same way as the IRE1α-FL vector, except that P2A linker was switched to GS linker.

For the construction of *Ire1α*-I642G and *Ire1α*-K907A point mutation vectors, the *Ire1α*-FL plasmid was used as a template, and the vectors were generated using the Toyobo SMK-101 kit. The primer sequences are listed in Supplementary Table S4.

### *In vitro* transcription and preparation of mRNAs for microinjections

To produce mRNA, vectors were linearized using restriction enzymes. The linearized DNA was transcribed into RNA using the T7 mMACHINE Kit (Invitrogen, AM1340) following the manufacturer’s instructions. Poly(A) tails were added to the RNA’s 3′ ends using a poly(A) tailing kit (Invitrogen, AM1350). The targeted RNA (500 ng/μl) was microinjected into the cytoplasm of GV oocytes or PN1 zygotes by microinjector. Injected oocytes or embryos were cultured in M2 or KSOM medium at 37°C in a 5% CO_2_ incubator.

### Western blot

Approximately 100 embryos were lysed in SDS loading buffer at 95°C for 10 min. Total proteins were separated by sodium dodecyl sulfate–polyacrylamide gel electrophoresis (SDS–PAGE) and transferred onto polyvinylidene fluoride (PVDF) membranes. The membranes were blocked with 5% bovine serum albumin (BSA) in tris buffered saline tween (TBST) at room temperature (RT) for 1 h, followed by incubation with primary antibodies overnight at 4°C. After washing the membranes four times with TBST (10 min per time), they were incubated with HRP-conjugated secondary antibodies for 1 h at RT, and then washed three more times. Protein levels were visualized using chemiluminescence detection. A list of primary antibodies used is provided in antibodies section.

### *In vitro* RNA cleavage assay

The selected RNA was transcribed *in vitro* as described above. In cleavage assays, RNA (800 ng) was incubated with Recombinant Human IRE1α (aa 465–977; Abclonal, RP02121LQ) in the presence or absence of 10 μM 4μ8c for 1 h at 37°C. The reaction was carried out in a buffer containing 40 mM HEPES (pH 7.5), 2 mM Mg(OAc)_2_, 100 mM KOAc, and 1 mM dithiothreitol (DTT) [23]. Digestion products were separated on a 0.5% TBE–urea PAGE gel (Beyotime, R0231S) at 160 V for 1.5 h. The gel was stained in DEPC-treated water containing nucleic acid dye (Beyotime, D0145) for 30 min, and visualized under UV light.

For RT-PCR assay, mixed RNA (20 ng per gene) was digested with hIRE1α as described above. The digestion products were reverse-transcribed into cDNA (Transgen, AU341), which was then used as a template for RT-PCR.

### Immunofluorescent staining

Zygotes were fixed in 4% paraformaldehyde (PFA) for 30 min at RT, and then permeabilized with 0.5% Triton X-100 at RT for 20 min. After blocking in 1% BSA for 1 h at RT, the zygotes were incubated with primary antibody overnight at 4°C. The following day, the samples were washed four times (5 min each) in wash buffer (0.1% Tween 20 + 0.01% Triton X-100 in PBS). Secondary antibodies were applied for 2 h at RT, followed by four 10-min washes in wash buffer. 4',6-Diamidino-2-phenylindole dihydrochloride (DAPI) was added for nuclear staining, and samples were incubated for 30 min at RT. Zygotes were imaged using a Leica DMi8 confocal laser scanning microscope, and images were processed using ImageJ software (NIH).

### EU staining

Zygotes at hCG 24 h were cultured in KSOM medium containing 5 μM 5-ethynyl uridine (EU) for 2 h at 37°C, 5% CO_2_. They were then washed twice with PBS (5 min per wash at RT), fixed in PFA for 30 min at RT, and permeabilized with 0.5% Triton X-100 for 30 min. Subsequently, the samples were incubated with Apollo staining reaction solution (RiboBio, C10316-3) for 30 min at RT, washed four times with 0.5% Triton X-100 (10 min per wash), and then stained with DAPI. Observations were performed using the same protocol as for immunofluorescent staining.

### JC-1 and ROS staining

Zygotes of WT or IRE1α-GKO were cultured in KSOM medium containing JC-1 dye (Beyotime, C2003S) or ROS dye (Beyotime, S0033S) for 30 min at 37°C, 5% CO_2_. They were then washed three times with PBS and observed as samples of immunofluorescent staining.

### RNA-seq library preparation

RNA-seq libraries were prepared using the Smart-seq2 protocol [26], as described previously. Pronuclear stage 5 (PN5) zygotes or late two-cells were washed three times in PBS containing 0.2% BSA and collected in lysis buffer with RNase inhibitor for library preparation.

### LACE-seq library preparation

To prepare samples for LACE-seq, the zona pellucida of 300 IRE1α-Flag zygotes was removed using Tyrode’s solution (Sigma, T1788). The zygotes were then cross-linked with 0.1% formaldehyde at RT for 10 min, and the reaction was quenched with 150 mM glycine for 10 min. LACE-seq libraries were generated according to the previously published protocol [27], using a Flag antibody for immunoprecipitation.

### RNA-seq data analysis

The sequencing reads were aligned to the mouse reference genome (mm9) using HISAT2 (v2.1.0). For gene-level quantification, FeatureCount (v2.0.1) was employed with Gencode mm9 annotations as the reference. Read count normalization and differential expression analysis were performed using the DESeq2 package, where size factors were estimated prior to differential testing.

### LACE-seq data analysis

The raw sequencing reads were processed to remove adapter sequences at both ends using Cutadapt (v1.15) with the following parameters: -m 18 -j 8 --max-n 4 --trim-n --times 2 -e 0.1 -O 3. The adapter sequences for parameters -a, -A, and -G are provided in Supplementary Table S5. Paired-end reads with overlaps exceeding 30 nucleotides were merged into single reads using fastp (v0.21.0). Following UMI sequence extraction, the cleaned reads underwent a two-stage alignment process. First, reads were aligned to mouse pre-rRNA using Bowtie2 (v2.5.1). Unmapped reads were then aligned to the mouse reference genome (mm9) using STAR (v2.5.2b) with specific settings: --outSJfilterReads Unique --alignEndsType Extend5pOfRead1 --outFilterMismatchNoverLmax 0.04 --outFilterMismatchNmax 999 --outFilterMultimapNmax 1. PCR duplicates were removed using UMI information, and the unique reads were used for peak identification with PureCLIP (v1.3.1). Inter-replicate correlation was assessed using Pearson correlation coefficients, as previously described.

## Results

### IRE1α knockout in female mice causes female infertility

**Figure 1.**
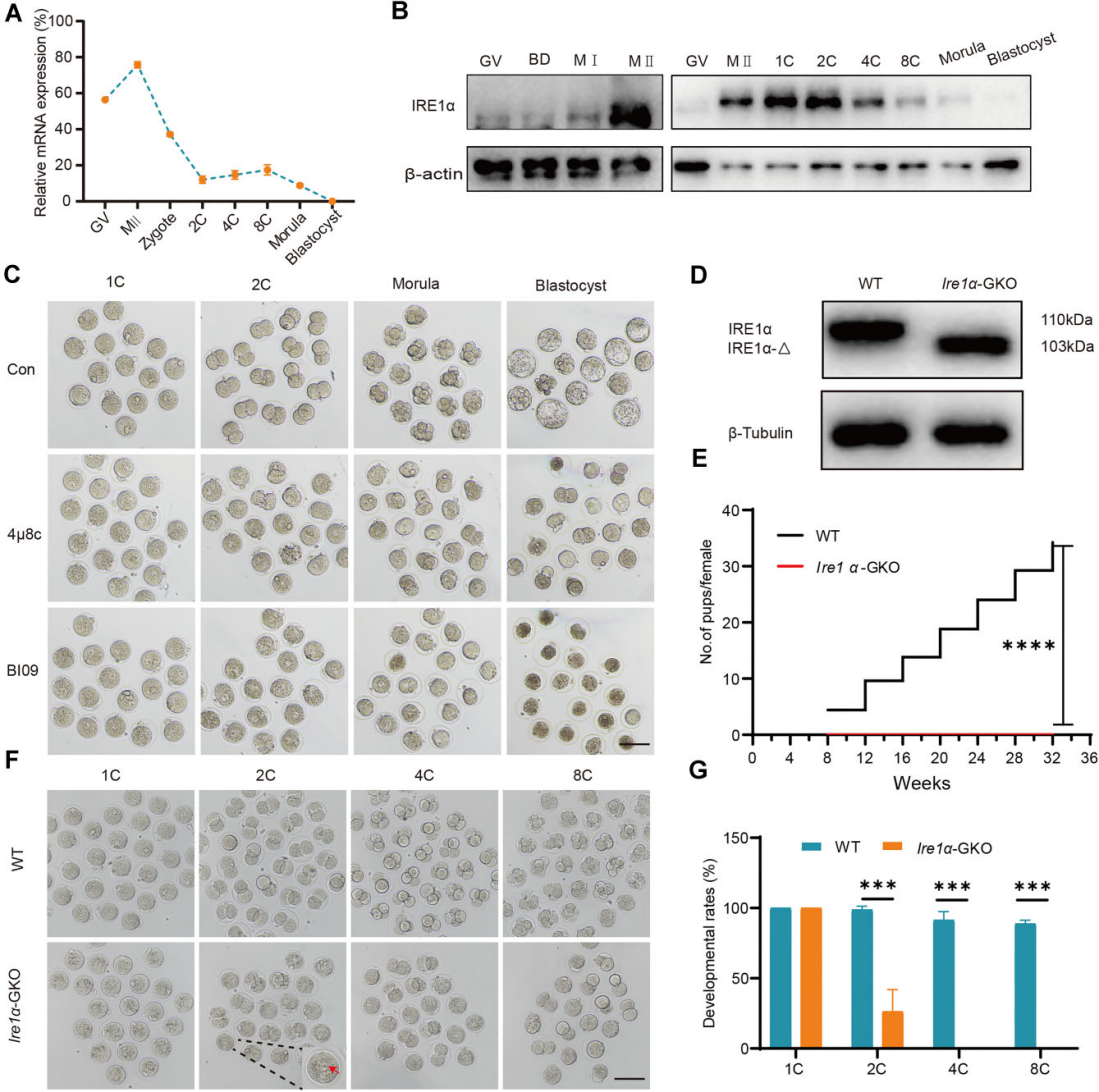
Embryos derived from IRE1α-GKO female mice were developmentally arrested. (**A**) RT-PCR results showing mRNA level of *Ire1α* in mouse oocytes and preimplantation embryos. (**B**) Western blot showing the protein level of IRE1α in mouse oocytes and preimplantation embryos. (**C**) Representative images of embryos treated with DMSO, 4μ8c (10 μM), or BI09 (10 μM) after hCG 18 h. Scale bar, 100 μm. (**D**) Western blot results showing the protein levels of IRE1α in zygotes of WT and *Ire1α*-GKO females. (**E**) Average number of offspring produced per WT and *Ire1α*-GKO females. The number of mice used: *n* = 5; ***P < .001 by two-tailed Student’s *t*-test. (**F**) Representative images of embryos derived from WT and IRE1α-GKO females. The red arrow indicates the pronuclear in the IRE1α-GKO embryo. Scale bar, 100 μm. (**G**) Developmental rates of embryos derived from WT and IRE1α-GKO females: WT, *n* = 43; *Ire1α*-GKO, *n* = 40; error bars, ***P < .001 by two-tailed Student’s *t*-test.

We first characterized the dynamic mRNA expression patterns in early mouse embryos. *Ire1α* mRNA was highly expressed in oocytes and zygotes, gradually declining until the blastocyst stage [28] (Supplementary Fig. S1A), which is confirmed by quantitative RT-PCR (Fig. 1A). Notably, the translation efficiency of *Ire1α* was highest in MII oocyte and zygote stages [28] (Supplementary Fig. S1B). Correspondingly, IRE1α protein level peaked during the one-cell and two-cell stages before declining at the four-cell stage (Fig. 1B), suggesting a potential maternal regulatory function after fertilization. Given that IRE1α primarily functions through its RNase domain, we initially treated early embryos with IRE1α RNase inhibitors 4μ8c and BI09, and found that the treated embryos arrested at the one-cell stage, indicating a specific and critical role of IRE1α in early embryogenesis (Fig. 1C and Supplementary Fig. S1C).

To comprehensively explore IRE1α’s physiological functions, we generated an oocyte-specific knockout mouse model using Cre-loxP system. By strategically excising exons 20 and 21 (encoding the RNase domain) via GDF9-cre without inducing a frameshift mutation, we created an oocyte-specific IRE1α RNase domain-deficient mouse line (Supplementary Fig. S1D). PCR identified the *Ire1α^fl/fl^;Gcre* (*Ire1α*-GKO) mouse had smaller gene amplification bands than that of WT mouse (Supplementary Fig. S1E). Since IRE1α is highly expressed at the zygote stage, we detected the protein expression level of IRE1α in *Ire1α*-GKO zygotes by western blot, and confirmed successful RNase domain deletion of IRE1α (IRE1α-Δ) without compromising overall protein expression in zygotes (Fig. 1D). In addition, the deletion of the RNase domain did not affect the location of IRE1α in zygotes (Supplementary Fig. S1F). Over 6 months for fertility tests, we found that the *Ire1α*-GKO females did not produce any offspring (Fig. 1E), suggesting that *Ire1α*-GKO females were completely infertile. We next cultured zygotes derived from *Ire1α*-GKO females *in vitro*, and found that most *Ire1α*-GKO embryos were arrested at the one-cell stage, with few reaching to the two-cell stage and none progressing to the four-cell stage. In contrast, WT embryos typically developed to two-cell and four-cell stages at corresponding time, highlighting IRE1α’s indispensable role in female reproductive potential as a maternal factor (Fig. 1F and G). To determine the developmental stage at which *Ire1α*-GKO embryos arrest, we performed EdU staining and observed that DNA replication proceeded normally (Supplementary Fig. S1G), indicating successful entry into the S phase. However, embryonic development was arrested at the one-cell stage with failed pronuclear fusion (Fig. 1F). These results collectively suggest that the developmental arrest occurs at the G2 phase. Interestingly, consistent with IRE1α's low expression in oocytes, implying little function in the oocyte maturation, these knockout oocytes maintained normal capabilities for GV breakdown and polar body extrusion (Supplementary Fig. S1H). Furthermore, *Ire1α* knockout mice ovulated similar number of MII oocytes by superovulation as WT mice (Supplementary Fig. S1I), and exhibited typical spindle morphology with neatly aligned chromosomes (Supplementary Fig. S1J). Taken together, these results show that IRE1α acts as a maternal factor regulating early embryonic development and its deletion causes early embryonic developmental arrest.

### The IRE1α RNase domain deletion neither affects mitochondrial function nor activates UPR pathways

Considering IRE1α is known to modulate function of mitochondria-associated membranes (MAMs) [29], we expect that embryo development arrest may be caused by mitochondrial dysfunction. We thus comprehensively analyzed mitochondrial dynamics following IRE1α deletion. Contrary to potential expectations, we observed that mitochondrial membrane potential was maintained at normal levels in IRE1α-deleted embryos (Supplementary Fig. S2A and B). The reactive oxygen species (ROS) was unexpectedly decreased in IRE1α-deleted embryos, suggesting no significant mitochondrial dysfunction (Supplementary Fig. S2C and D). These suggested IRE1α knockout did not affect mitochondrial function in zygotes.

**Figure 2.**
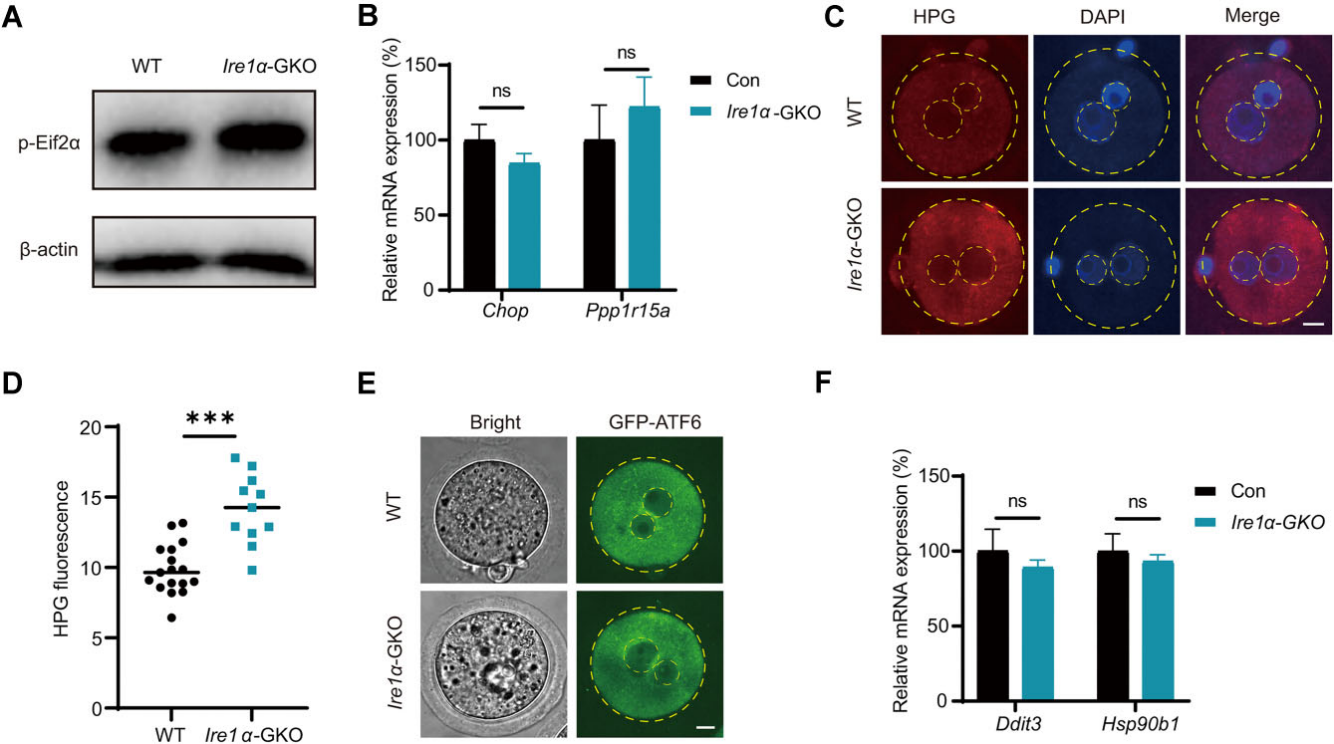
The IRE1α RNase domain deletion does not activate UPR pathways. (**A**) Western blot showing the phosphorylation level of eIF2α in WT and *Ire1α*-GKO zygotes. (**B**) RT-qPCR validation of *Chop* and *Ppp1r15a* in WT and *Ire1α*-GKO zygotes. (**C**) HPG staining showing the newly translated protein in WT and *Ire1α*-GKO zygotes. Scale bar, 10 μm. (**D**) HPG fluorescence intensity statistics of WT and *Ire1α*-GKO zygotes. ***P <.001; *P*-values were determined by two-tailed unpaired *t*-test. (**E**) Fluorescence microscopy results showing the location of GFP-ATF6. Scale bar, 10 μm. (**F**) RT-PCR showing the mRNA levels of *Ddit3* and *Hsp90b1* in WT and *Ire1α*-GKO zygotes.

The UPR represents a complex cellular stress mechanism with three interconnected pathways: IRE1α, PERK, and ATF6 [25, 30]. To definitively understand the mechanism of embryonic developmental arrest, we further conducted an exhaustive investigation of these pathways. Activated PERK phosphorylates translation initiation factor eIF2α (p-eIF2α) and thus inhibits most mRNA translation, and p-eIF2α enhances the translation of transcription factor ATF4, which regulates transcription of downstream genes. Therefore, we measured the phosphorylated eIF2α protein level in *Ire1α*-GKO zygotes to test whether the embryonic developmental arrest is due to the altered eIF2α phosphorylation. However, we find the level of eIF2α phosphorylation remained unchanged in *Ire1α*-GKO zygotes (Fig. 2A), and the relative mRNA levels of ATF4 downstream targets (*Chop* and *Ppp1r15a*) were not significant alterations (Fig. 2B). In contrast, HPG staining, which characterizes the newly synthesized protein, revealed an unexpected increase in protein synthesis, contrary to expected translational inhibition in *Ire1α*-GKO embryos (Fig. 2C and D). When ATF6 is activated, it is transported to the Golgi apparatus and then cut into two fragments, with the N-terminal forming an active transcription factor that promotes the expression of UPR-related genes. Since no suitable ATF6 antibody was found for immunofluorescence and western blot in zygotes, we employed an innovative approach with GFP tagged to the N-terminal of ATF6 and injected its mRNA into the zygote. We found that *Ire1α* deletion did not increase nuclear translocation of ATF6 transcription factor (Fig. 2E). Similarly, mRNA levels of ATF6 target genes *Ddit3* and *Hsp90b1* were unchanged (Fig. 2F). Our systematic analysis conclusively ruled out the possibility that embryonic developmental arrest results from compensatory activation of alternative UPR signaling pathways. This finding is particularly significant as it challenges conventional understanding of UPR pathway interactions, and suggests a unique, UPR pathway-independent mechanism of IRE1α function in early embryogenesis.

### The dysfunction of IRE1α RNase is the main cause of embryonic developmental arrest

**Figure 3.**
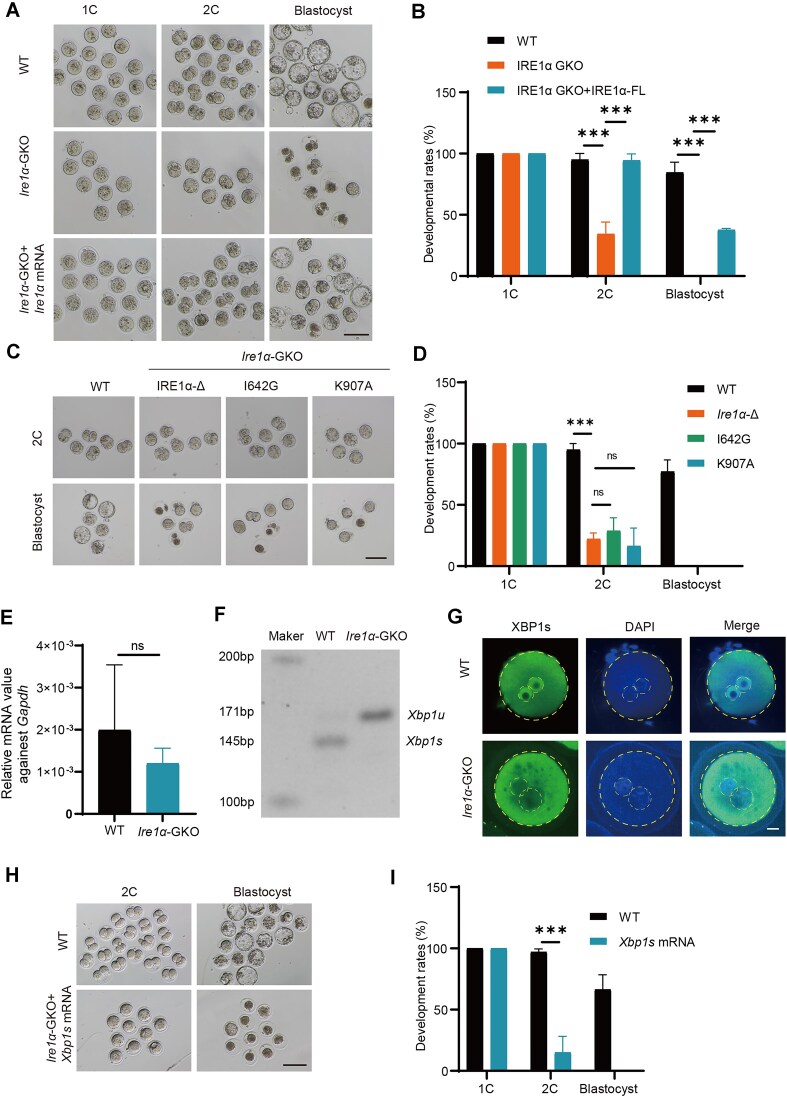
IRE1α RNase activity is important for early embryonic development. (**A**) Representative images of WT, *Ire1α*-GKO, and *Ire1α*-GKO + *Ire1α* mRNA (microinjection of *Ire1α* mRNA in *Ire1α*-GKO zygotes) embryos. Scale bar, 100 μm. (**B**) Statistics of the proportion of WT, *Ire1α*-GKO, and *Ire1α*-GKO + *Ire1α* mRNA embryo development when WT embryos reached the corresponding stages. ***P < .001, as assessed by two-tailed Student’s *t*-test. (**C**) Representative images of WT, *Ire1α*-Δ (microinjection of *Ire1α*-Δ mRNA in *Ire1α*-GKO zygotes), I642G (microinjection of *Ire1α*-I642G mRNA in *Ire1α*-GKO zygotes), and K907A (microinjection of *Ire1α*-K907A mRNA in *Ire1α*-GKO zygotes) embryos. Scale bar, 100 μm. (**D**) Statistics of the proportion of WT, *Ire1α*-Δ, I642G, and K907A embryo development when WT embryos reached the corresponding stages. ***P < .001, as assessed by two-tailed Student’s *t*-test. (**E**) RT-PCR showing the mRNA levels of *Xbp1* in WT and *Ire1α*-GKO zygotes. (**F**) PCR showing *Xbp1u* and *Xbp1s* in WT and *Ire1α*-GKO zygotes. (**G**) immunofluorescent staining showing the XBP1s in WT and *Ire1α*-GKO zygotes. (**H**) Representative images of WT and *Ire1α*-GKO + *Xbp1s* mRNA (microinjection of *Xbp1s* in *Ire1α*-GKO zygotes) embryos. Scale bar, 100 μm. (**I**) Statistics of the proportion of WT and *Ire1α*-GKO + *Xbp1s* mRNA embryo development when WT embryos reached the corresponding stages. ***P < .001, as assessed by two-tailed Student’s *t*-test.

As a maternal factor, IRE1α was specifically expressed highly in the one-cell and two-cell stages. We hypothesized that restoring IRE1α in *Ire1α*-GKO embryos would rescue their developmental arrest. To confirm the expression of *Ire1α* mRNA after microinjection by using a fluorescent protein, and ensuring that the fluorescent protein does not interfere with the structure or function of IRE1α, we added P2A and the fluorescent protein mCherry sequence to the C-terminus of IRE1α (IRE1α-P2A-mCherry, hereafter referred to as IRE1α-FL) (Supplementary Fig. S3A), and the mRNA was microinjected into the zygote, which could be expressed normally (Supplementary Fig. S3B). As expected, when we supplemented *Ire1α-*FL mRNA in *Ire1α*-GKO zygotes via microinjection, all *Ire1α*-GKO zygotes progressed to the two-cell stage, with some even developing to the blastocyst stage (Fig. 3A and B). This finding suggested that IRE1α plays a crucial role specifically during early embryonic stages after fertilization. We further microinjected *Ire1α* mRNA into *Ire1α*-GKO zygotes at different time points. The results showed that while most embryos successfully developed to the two-cell stage, the blastocyst rate progressively decreased with delayed injection timing. These findings indicate that IRE1α begins to exert its functional role during the early zygotic stage (Supplementary Fig. S3C and D). Beyond its canonical function as a UPR signal transducer, IRE1α also serves as a scaffold protein that facilitates InsP3R docking at MAMs, a process dependent on its structural integrity [29]. To determine whether IRE1α functions through its structural configuration or RNase activity, we microinjected *Ire1α*-I642G (kinase dead, also induced RNase dead) and *Ire1α*-K907A (RNase dead only) mRNA into *Ire1α*-GKO zygotes to rescue the phenotype of embryonic developmental arrest (Supplementary Fig. S3E). Notably, neither *Ire1α*-I642G nor *Ire1α*-K907A could rescue the *Ire1α* deficient phenotype (Fig. 3C and D). These results strongly indicated that *Ire1α* functions in zygotes primarily through its RNase activity rather than its protein structure.

*Xbp1* is the classic substrate of the IRE1α RNase domain, which can be spliced to generate *Xbp1s* RNA, subsequently translated into an active transcription factor. We first examined the RNA levels of *Xbp1* and found that although its expression relative to *Gapdh* was very low, its RNA levels remained unaffected in *Ire1α*-GKO zygotes (Fig. 3E). Further studies revealed that *Xbp1* mRNA completely failed to undergo splicing (Fig. 3F). Additionally, there was no XBP1s protein detected in *Ire1α*-GKO mouse embryos (Fig. 3G). These results suggest that the IRE1α–XBP1 pathway may play a critical role in early embryonic development. Previous research in medaka fish demonstrated that the IRE1α–XBP1 pathway regulates early embryonic development [31]. So, we microinjected *Xbp1s* mRNA into *Ire1α*-GKO zygotes, but the zygotes remained arrested at the one-cell stage, and only a small proportion developed to the two-cell stage, indicating that XBP1s could not rescue the phenotype resulting from IRE1α deletion (Fig. 3H and I). Additionally, the mRNA levels of XBP1 target genes *Dnajb9* and *Pdia3* showed no significant decrease in *Ire1α*-GKO zygotes compared to WT zygotes (Supplementary Fig. S3F). Furthermore, the mRNA level and translation efficiency of *Xbp1* remained low during early embryonic stages, only increasing substantially at the blastocyst stage—an expression pattern markedly different from that of *Ire1α* [28] (Supplementary Fig. S3G and H). Collectively, these results conclusively demonstrate that IRE1α functions in zygotes through its RNase activity, independent of the canonical IRE1α–XBP1 signaling pathway.

### IRE1α RNase domain regulates maternal mRNA decay

**Figure 4.**
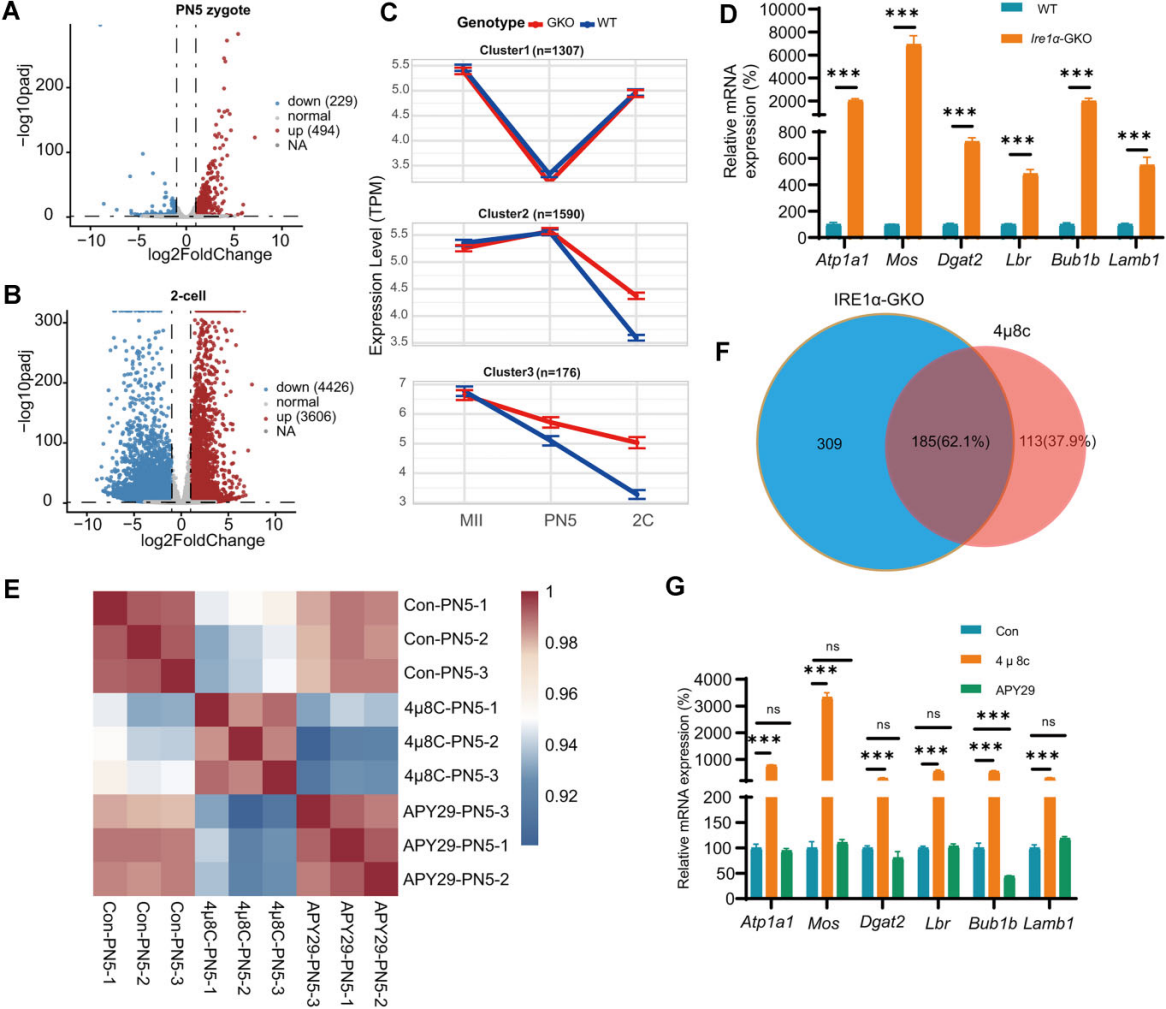
IRE1α regulates maternal mRNA decay. Volcano plot showing transcriptome changes in *Ire1α* knockout PN5 zygotes (**A**) and two-cell (**B**). (**C**) The degradation pattern of maternal transcripts in mouse embryos derived from WT and *Ire1α*-GKO females. (**D**) RT-PCR showing the relative RNA expression of upregulated transcripts in *Ire1α*-GKO PN5 zygotes compared to WT PN5 zygotes. ***P< .001, as assessed by two-tailed Student’s *t*-tests. (**E**) Heat map of Spearman correlation coefficients among control group, 4μ8c (10 μM) group, and APY29 (10 μM) group of PN5 zygotes. (**F**) Venn diagrams showing the overlap of upregulated transcripts in mouse PN5 zygotes with or without 4μ8c treatment. (**G**) RT-PCR showing the relative mRNA expression of transcripts of PN5 zygotes treated with 4μ8c (10 μM) and APY29 (10 μM). ***P < .001, as assessed by two-tailed Student’s *t*-tests.

Beyond the IRE1α–XBP1 pathway, the IRE1α RNase domain can directly cleave RNA through a mechanism known as RIDD, with multiple RNAs previously identified as IRE1α direct binding and cleavage targets. To investigate the effects of IRE1α deletion on mRNA metabolism during MZT, we analyzed the RNA-seq data of WT and *Ire1α*-GKO PN5 zygotes. We detected over 10 000 genes (FPKM(Fragments per kilobase of exon model per million mapped fragments) > 0.1) across all samples; as expected, RNA fragments encoded by *Ire1α* exons 20 and 21 were completely absent in *Ire1α*-GKO zygotes, while other fragments were expressed normally (Supplementary Fig. S4A). We detected high correlation of transcripts confirmed by principal component analysis (Supplementary Fig. S4B). Compared to the WT group, *Ire1α* depletion in PN5 zygotes and two-cell resulted in a substantial number of differentially expressed genes (DEGs) (Fig. 4A and B). However, the DEGs in GV and MII oocytes between WT and *Ire1α*-GKO were little affected (Supplementary Fig. S4C and D), which is consistent with the normal phenotype of *Ire1α*-GKO MII oocyte (Supplementary Fig. S1G–I).

Given that maternal mRNA continues to degrade after fertilization and IRE1α specifically functioned after fertilization through its RNase domain, we hypothesized that IRE1α might regulate maternal mRNA degradation. By analyzing gene expression patterns in MII, PN5 zygote, and two-cell, we divided transcripts into six clusters: cluster 1, comprising transcripts that were substantially degraded at the MII–PN5 transition and subsequently stably expressed or re-accumulated at the two-cell stage; cluster 2, comprising transcripts that stably expressed at MII–PN5 transition, and degraded at the PN5–two-cell transition; cluster 3, comprising transcripts that were degraded at the MII–two-cell transition; cluster 4, comprising transcripts that were stable at the MII–two-cell transition; cluster 5, comprising transcripts that were substantially upregulated at the MII–PN5 transition; and cluster 6, comprising transcripts that were substantially upregulated at the PN5–two-cell transition. Cluster 1 and cluster 3 were considered to be maternal mRNAs degraded in zygote stages, and cluster 2 and cluster 3 were considered to be maternal mRNAs degraded in two-cell stages. Cluster 5 was considered to be a minor ZGA RNA, and cluster 6 was considered to be a major ZGA RNA (Supplementary Fig. S4E). By analyzing transcripts in *Ire1α*-GKO embryos, we found that the maternal mRNAs of cluster 2 and cluster 3 were stable at zygote and two-cell stage (Fig. 4C). Given that deletion of IRE1α RNase domain mainly resulted in embryonic developmental arrest at the one-cell stage, we further detected the degradation of maternal mRNA at PN5 stage; RT-PCR confirmed that these transcripts were upregulated in *Ire1α*-GKO compared to WT zygotes (Fig. 4D). Considering the endonuclease function of IRE1α, these results implied that IRE1α may selectively degrade many maternal mRNAs at zygote and two-cell stages.

To further investigate the importance of IRE1α RNase domain in maternal mRNA degradation, we treated zygotes with two distinct IRE1α inhibitors: 4μ8c (which inhibit IRE1α RNase activity) and APY29 (a kinase inhibitor that does not affect IRE1α RNase activity) separately. 4μ8c was employed to validate the *Ire1α*-GKO phenotype through RNase enzymatic blockade, while APY29 served as a negative control to investigate RNA-mediated regulation of maternal mRNA degradation. As expected, 4μ8c treatment resulted in impaired degradation of a large amount of maternal mRNAs (296), while APY29 treatment minimally affected transcript levels (16) (Supplementary Fig. S4F and G). This result strongly supports the central role of IRE1α RNase activity in RNA decay. We analyzed the correlation of top 2000 transcripts of the control group, 4μ8c group, and APY29 group, and found that the APY29 group had a higher correlation with control than the 4μ8c group (Fig. 4E), and 62.1% transcripts upregulated in the 4μ8c group showed impaired degradation in *Ire1α*-GKO zygotes (Fig. 4F). RT-PCR further validated that the maternal mRNAs were degraded in the APY29 group, but stabilized in the 4μ8c group (Fig. 4G). These results showed that the maternal mRNA degradation was dependent on IRE1α RNase activity.

In our results, we also found that both minor and major ZGA were affected in *Ire1α*-GKO zygote and two-cell (Supplementary Fig. S5A). Previous research has established that ZGA is crucial for maternal mRNA clearance [11, 32, 33]. We labeled newly synthesized mRNAs with EU, which were cultured with zygotes for 2 h. Strong EU signals were detected in the nuclei of WT zygotes but not in IRE1α-GKO zygotes (Supplementary Fig. S5B), and the fluorescence signal intensity of EU in the IRE1α-GKO zygotes was much lower than that in WT zygotes (Supplementary Fig. S5C). Consistently, the phosphorylation of the second serine in the carboxyl-terminal domain (CTD) of RNA polymerase II was also affected (Supplementary Fig. S5D and E). Since zygotes lacking IRE1α RNase activity also showed partially decreased transcription levels, we investigated whether abnormal ZGA might explain the aberrant maternal mRNA degradation. When zygotes were treated with the transcription inhibitor DRB alone, maternal transcripts degraded normally; however, when 4μ8c was added, the degradation of the maternal transcripts was inhibited (Supplementary Fig. S5F). These results indicate that the dysregulation of maternal mRNA degradation was not caused by abnormalities of minor ZGA. We then treated embryos during one- and two-cell stages with DRB, and found there were many upregulated transcripts at late two-cell stage (Supplementary Fig. S5G). However, there were only 34% transcripts upregulated in DRB treatment overlapped with *Ire1α*-GKO two-cell embryos (Supplementary Fig. S5H), which can only partially explain the impaired degradation of maternal mRNAs caused by IRE1α deletion. Collectively, these results provide compelling evidence that IRE1α is responsible for maternal mRNA degradation through its RNase activity independent of ZGA.

### IRE1α directly cleaves maternal mRNAs *in vitro*

As an RNA-binding protein, IRE1α plays a critical role in maternal mRNA decay during early embryonic development. To investigate whether IRE1α directly binds and degrades maternal mRNAs, we utilized LACE-seq to identify RNAs bound by IRE1α. To achieve this, we generated an IRE1α-Flag knock-in mouse model, where the Flag epitope was linked to the C-terminal of IRE1α (Supplementary Fig. S6A). To ensure that the FLAG-tagged IRE1α did not interfere with its structure and function, we injected *Ire1α*-Flag mRNA into *Ire1α*-GKO zygotes. The injection of IRE1α-Flag mRNA rescued the developmental arrest observed in *Ire1α*-GKO embryos (Supplementary Fig. S6B and C), similar to the phenomenon seen in *Ire1α*-FL mRNA rescued assay (Fig. 3A), indicating that the FLAG-tag did not disrupt IRE1α’s functional integrity. Subsequently, we used anti-FLAG antibodies to immunoprecipitate IRE1α-Flag protein, allowing us to construct a library of RNAs directly bound by IRE1α. This approach helped us identify the maternal mRNAs that IRE1α binds and potentially regulates.

**Figure 5.**
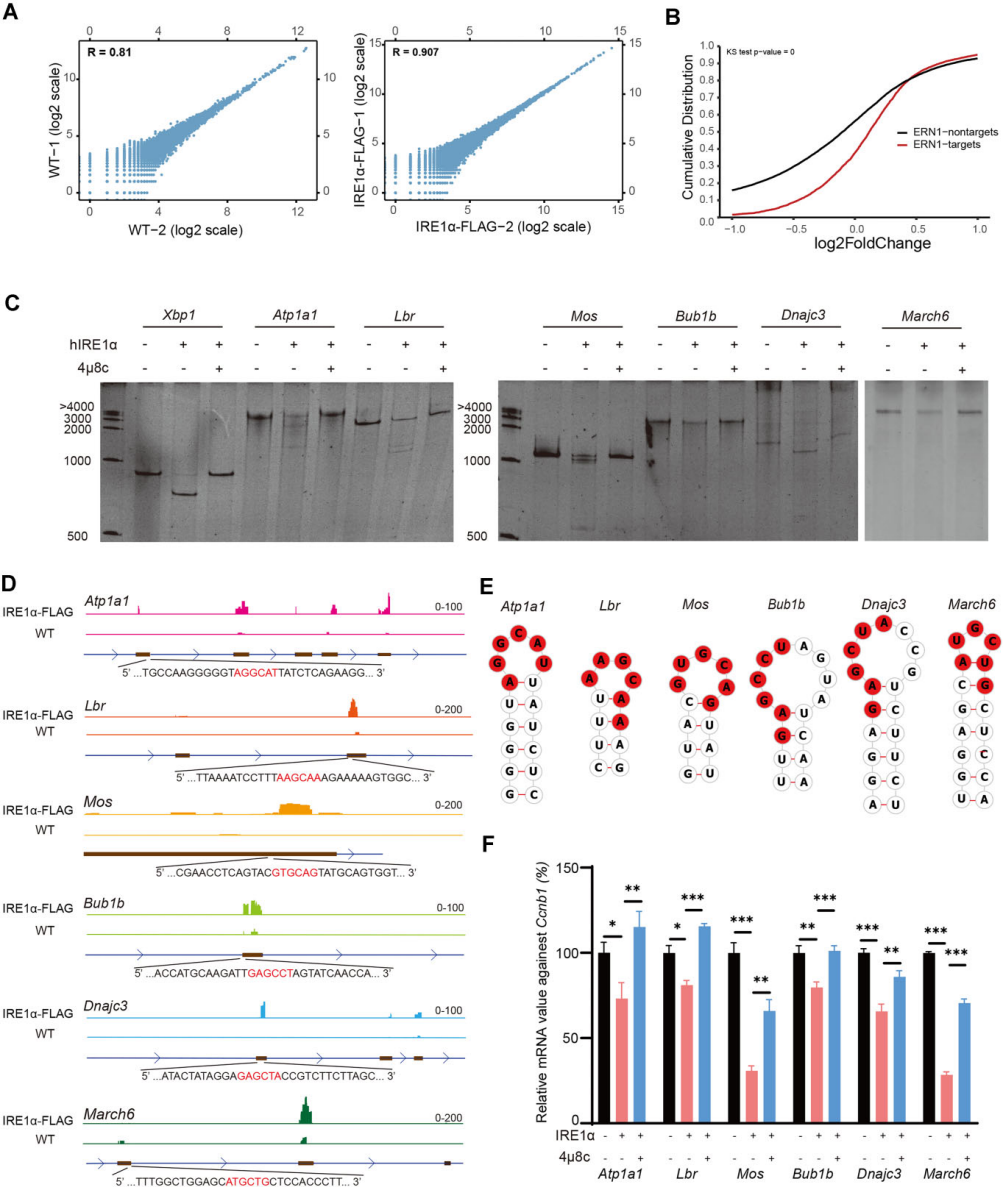
IRE1α directly cleaves maternal mRNAs. (**A**) Scatter plots showing the correlation between biological replicates of anti-IgG and anti-FLAG group (Pearson correlation coefficients). (**B**) Cumulative distribution function plot showing the transcript level changes after IRE1α depletion in zygotes. *P*-values were calculated by a two-tailed Kolmogorov–Smirnov test. (**C**) Urea-PAGE showing cleaved RNA fragments after treating with recombinant hIRE1α protein and 4μ8c *in vitro*. (**D**) Genome browser snapshot showing the read coverage of the gene locus in IRE1α-Flag LACE-seq data. The nucleotide sequences indicat potential cleavage sites in RNA. Red bases indicate motifs recognized by IRE1α. (**E**) Hairpin structures formed by potential cleavage sites. Red bases indicate motifs recognized by IRE1α. (**F**) RT-PCR showing the potential cleavage sites cleaved by IRE1α *in vitro*. The base sequence with a red background represents the IRE1α recognition motif.*P <.05, **P <.01, and ***P <.001 as assessed by two-tailed Student’s *t*-tests.

We collected embryos derived from WT and IRE1α-Flag knock-in mice at hCG 24 h, and constructed LACE-seq libraries using FLAG antibody. The power of LACE-seq was demonstrated by its high reproducibility between biological replicates (*R* > 0.8) (Fig. 5A). We divided all the detectable transcripts into two groups based on mapped IRE1α binding events. Compared with the IRE1α nontarget group, the IRE1α-bound transcripts were preferably upregulated in IRE1α-null zygotes, and the binding strength positively correlated with the upregulated fold change (Fig. 5B). We further examined the transcript levels of RNAs directly bound by IRE1α in IRE1α-GKO embryos and found that the majority of RNAs that remained stable in the knockout embryos (cluster 2 and cluster 3) were directly bound by IRE1α (Supplementary Fig. S7A). These overlapping genes are likely to be directly regulated by IRE1α. It has been previously reported that IRE1α exhibits endonuclease activity and cleaves its target mRNAs directly *in vitro*. Based on this, we hypothesized that IRE1α might also directly degrade maternal mRNAs identified by LACE-seq in the early zygote stage. To test this, we focused on maternal mRNAs that were degraded in WT zygotes but not in *Ire1α*-GKO zygotes. These mRNAs were specifically pulled down by IRE1α, supporting the idea that IRE1α plays a direct role in their decay. Among the identified RNAs, we randomly selected several transcripts, including *Atp1a1*, *Lbr*, *Mos*, *Bub1b*, *Dnajc3*, and *March6*, for further analysis. Consistent with the results from both the transcriptomic and LACE-seq data, we found that recombinant human IRE1α (amino acids 465–977) cleaved these maternal mRNAs directly *in vitro* (Fig. 5C and Supplementary Fig. S7B). Furthermore, the addition of 4μ8c, a known inhibitor of IRE1α, completely blocked the cleavage of these mRNAs (Fig. 5C). In contrast, the *Ccnb1* and maternal mRNAs, such as *Efna1*, *Rab6a*, and *Pacd6*, that were normally degraded in *Ire1α*-GKO zygotes were refractory to IRE1α-mediated splicing *in vitro* (Supplementary Fig. S7C), confirming the specificity of the cleavage activity.

The RNase domain of IRE1α is known to function as an endonuclease that cleaves RNAs containing specific structural motifs, particularly those with a hairpin structure and the CUGCAG sequence. To explore the potential position cleaved by IRE1α, we found that IRE1α binds to multiple different positions of these mRNAs (Fig. 5D and Supplementary Fig. S7D). We performed *in silico* analysis of the secondary structures of the identified maternal mRNAs using the RNAfold web server [34]. This analysis revealed that all these mRNAs contained an *Xbp1*-like motif (5′-CUG↓CAG-3′) near the regions where IRE1α binds (Fig. 5D and Supplementary Fig. S7D). Furthermore, the secondary structures of these mRNAs included stem-loop formations similar to the one observed in the well-characterized IRE1α target, *Xbp1*. These motifs represent potential cleavage sites for IRE1α (Fig. 5E and Supplementary Fig. S7E). The predicted cleavage sites partially correspond to the observed band sizes in TBE–urea PAGE gel. For instance, *Atp1a1* RNA (3072 bp in length) showed cleavage bands at ∼1000 and 2000 bp, aligning with its predicted cleavage sites at positions 1008 (*Atp1a1*-s1) and 1985 (*Atp1a1*-s2). Similarly, for *Mos* RNA (1032 bp in length), a band slightly smaller than the full length was observed in gel, which aligns with the predicted cleavage site identified at position 849 in the *Mos* RNA sequence (Fig. 5C and Supplementary Fig. S7D and E). To further confirm these cleavage sites experimentally, we designed qPCR primers flanking the predicted cleavage sites and performed RT-PCR analysis. As expected, we observed a significant reduction in the amplification of fragments containing the potential cleavage sites in the presence of recombinant IRE1α, compared to the control gene *Ccnb1*, which was not cleaved by IRE1α *in vitro* (Fig. 5F and Supplementary Fig. S7F). Moreover, the addition of 4μ8c together with IRE1α effectively prevented the cleavage of these RNA fragments (Fig. 5F), further supporting the notion that IRE1α is directly responsible for the maternal mRNA degradation. Collectively, these results provide strong evidence that IRE1α directly cleaves maternal mRNAs *in vitro*. This direct interaction between IRE1α and its RNA targets, particularly those with *Xbp1*-like motifs and stem-loop structures, underscores the pivotal role of IRE1α in regulating maternal mRNA decay during early embryonic development.

### Aberrant maternal mRNA degradation leads to aberrant histone modification and DNA methylation

**Figure 6.**
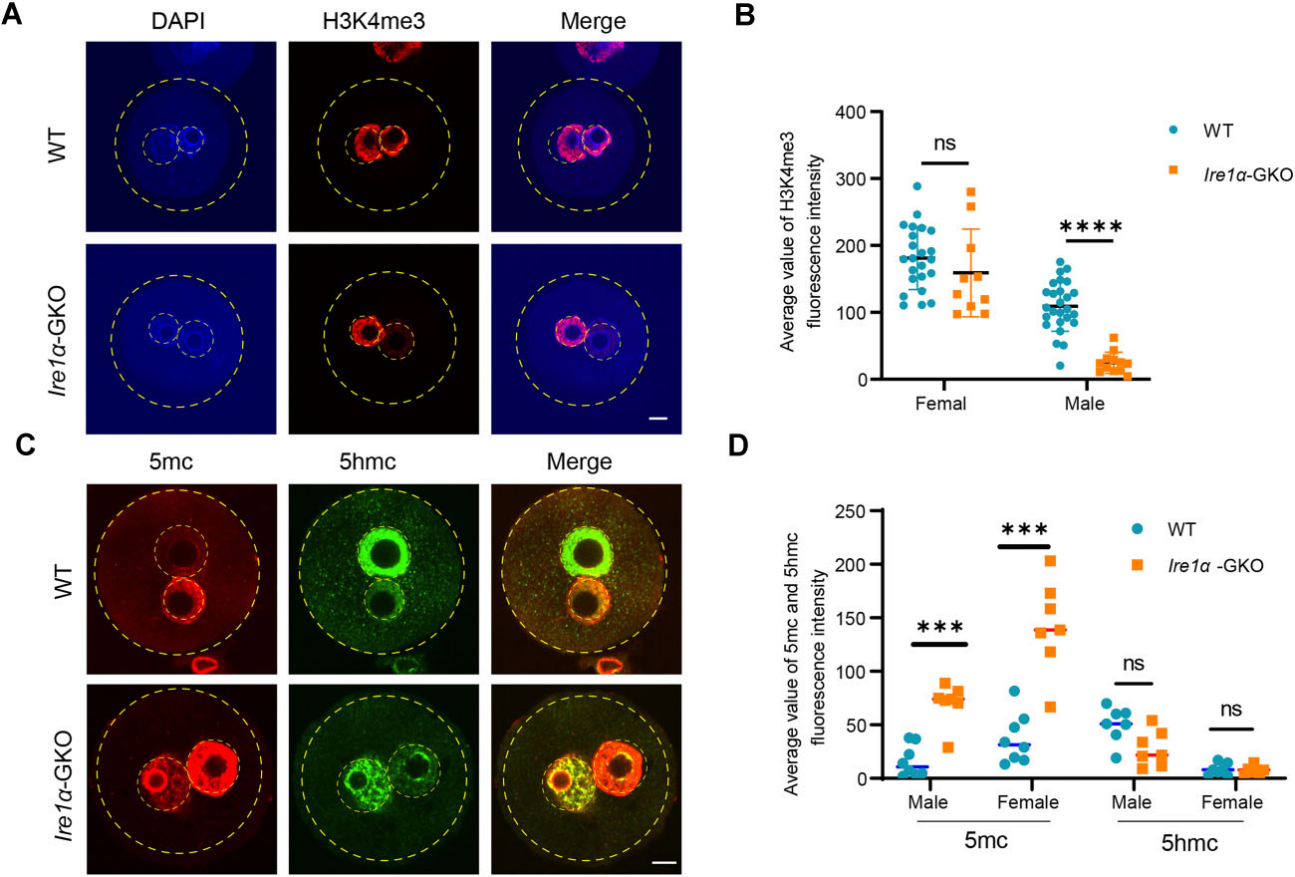
*Ire1α* depletion affects histone modification and DNA methylation. (**A**) Immunofluorescence staining of H3K4me3 and DNA in WT and *Ire1α*-GKO zygotes. Scale bar, 20 μm. (**B**) Fluorescence intensity statistics of Ser2p in WT and *Ire1α*-GKO zygotes. ***P < .001 by two-tailed Student’s *t*-tests. (**C**) Immunofluorescence staining of 5mc and 5hmc in WT and *Ire1α*-GKO zygotes. Scale bar, 20 μm. (**D**) Fluorescence intensity statistics of 5mc and 5hmc in WT and *Ire1α*-GKO zygotes. ***P < .001 by two-tailed Student’s *t*-tests.

In zygotes, histone modifications were reconstructed in pronuclear stage. Impaired RNA degradation leads to failed ZGA in IRE1α embryos. We asked whether IRE1α depletion would affect histone modifications and DNA methylation. In zygotes, the male pronucleus exhibits progressive enrichment of H3K4me3 [35, 36]. In this study, we examined the changes of H3K4me3 in male and female pronuclei, and found that female pronuclei were largely unaffected, while the level of H3K4me3 in male pronuclei was significantly reduced (Fig. 6A and B). The level of DNA methylation is also involved in the regulation of gene transcription, and DNA methylation levels progressively decrease in the maternal and paternal pronuclei of zygotes [37]. In our results, we found that the methylation level of DNA was abnormally increased in IRE1α-deficient zygotes (Fig. 6C and D), which may be one of the reasons for the decreased transcription. Collectively, IRE1α-regulated RNA decay is required to minor ZGA, histone modification, and DNA methylation changes that are essential for early embryo development.

### ERK1/2 couples IRE1α translation to oocyte maturation

**Figure 7.**
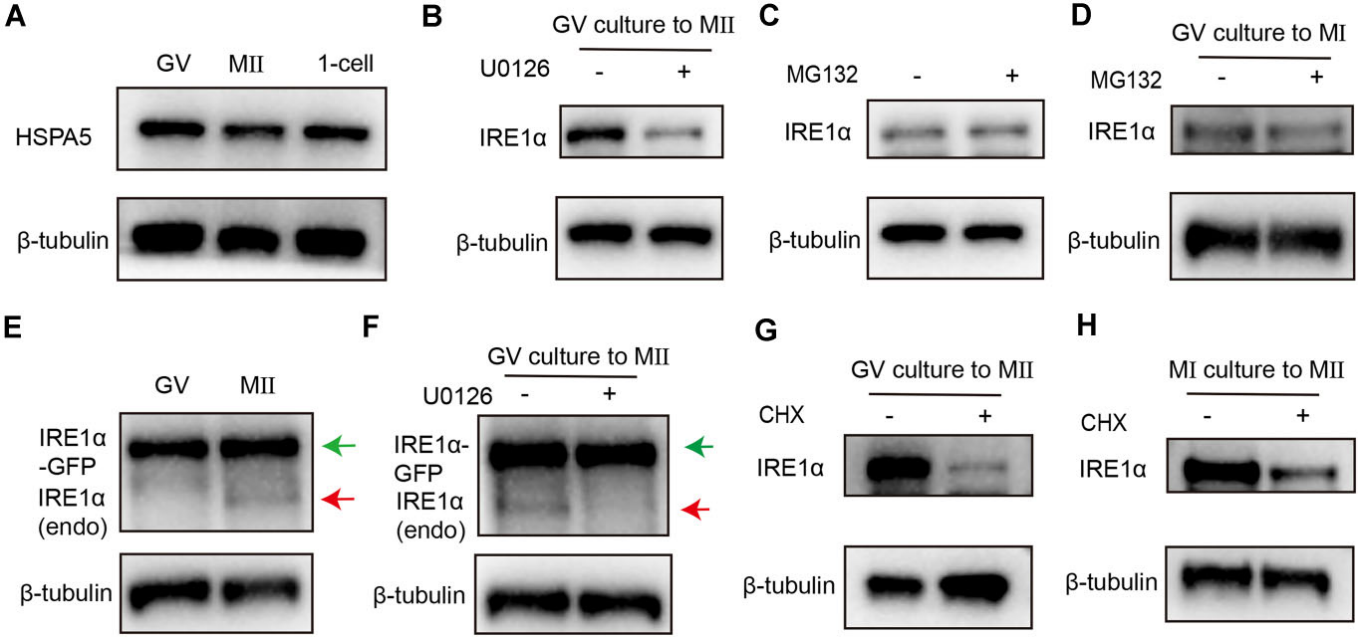
ERK1/2 triggers IRE1α mRNA translation during oocyte maturation. (**A**) Western blot results showing levels of HSPA5 in GV, MII oocytes, and one-cell. (**B**) Western blot results showing levels of IRE1α in MII oocytes with or without U0126 (20 μM) treatment. (**C**) Western blot results showing levels of IRE1α in GV oocytes with or without MG132 (10 μM) treatment. (**D**) Western blot results showing levels of IRE1α in MI oocytes with or without MG132 (10 μM) treatment. (**E**) Western blot results showing that injected mRNAs encoded for IRE1α-GFP were stably expressed in GV oocytes and MII oocytes. Red arrow: IRE1α; green arrow: IRE1α-GFP. (**F**) Western blot results showing levels of IRE1α-GFP in MII oocytes with or without U0126 (20 μM) treatment. Red arrow: IRE1α; green arrow: IRE1α-GFP. (**G**) Western blot results showing levels of IRE1α in MII oocytes with or without CHX (20 μM) treatment at GV stages. (**H**) Western blot results showing levels of IRE1α in MII oocytes with or without CHX (20 μM) treatment at MI stages.

The RNA level of *Ire1α* was high in GV oocytes, while its protein expression was significantly elevated in MII oocytes and reached its peak at the one-cell stage. Since IRE1α is an UPR protein that responds to ER stress, we examined the ER stress marker HSPA5 to evaluate ER stress levels in oocytes and embryos. Notably, the protein levels of HSPA5 did not increase during oocyte maturation and after fertilization (Fig. 7A), suggesting that the expression of IRE1α is not associated with ER stress in oocytes and embryos.

During meiotic maturation, ERK1/2 is phosphorylated and activated, and it has been reported to regulate maternal mRNA translation. To investigate this further, we treated oocytes with the ERK1/2 activation inhibitor U0126. Consistent with previous findings from our laboratory and others [9, 38, 39], we observed that it exerted minimal effects on meiotic progression during the GV–M2 stage (Supplementary Fig. S8A), and we found that the protein levels of IRE1α were significantly reduced in MII oocytes (Fig. 7B). Previous studies have indicated that ERK1/2 activation can prevent the degradation of IRE1α, leading us to hypothesize that the low levels of IRE1α in GV oocytes might be due to degradation. To test this, we treated GV oocytes with the proteasome inhibitor MG132 but observed no increase in IRE1α accumulation (Fig. 7C). Since MG132 impairs APC/C (anaphase-promoting complex/cyclosome) activity and prevents oocytes from progressing to the MII stage (Supplementary Fig. S8B), we collected oocytes treated with MG132 at the GV–MI stage, a developmental phase where IRE1α remains at low levels in WT oocytes (Fig. 1B). Notably, IRE1α expression did not increase under MG132 treatment (Fig. 7D), demonstrating that the low abundance of IRE1α during the GV–MI stage is not mediated by proteasomal degradation. Then we microinjected *Ire1α*-GFP mRNA into oocytes and treated them with or without milrinone. We observed that IRE1α-GFP was stably expressed in both GV and MII stage oocytes (Fig. 7E and Supplementary Fig. S8C). And when oocytes were treated with U0126, the exogenous IRE1α-GFP protein was stably expressed, while only the expression of endogenous IRE1α protein was suppressed (Fig. 7B and F). These results suggest that the translation, rather than the degradation, of IRE1α is regulated by ERK1/2. And this was further confirmed by treating oocytes with the protein synthesis inhibitor cycloheximide (CHX), which prevented the accumulation of IRE1α protein during oocyte maturation (Fig. 7G). Considering that CHX disrupts oocyte meiotic progression (Supplementary Fig. S8D) and that IRE1α begins to be highly expressed during the MI–MII transition (Fig. 1B), we treated oocytes with CHX at the MI stage (Supplementary Fig. S8E). Notably, these oocytes developed normally to the MII stage (Supplementary Fig. S8F), yet exhibited reduced IRE1α protein levels (Fig. 7H). These demonstrate that the reduction in IRE1α levels caused by CHX is attributable to translational inhibition. In summary, our findings suggest that ERK1/2 plays a crucial role in regulating the translation of IRE1α during oocyte maturation.

## Discussion

IRE1α, an ER transmembrane protein, plays a critical role in maintaining ER homeostasis through the IRE1α–XBP1 and RIDD pathways. Beyond its canonical functions, IRE1α acts as an essential RNA-binding protein, exerting its influence through RNA cleavage activity in various physiological processes. In mice, the disruption of IRE1α function precipitates extensive developmental anomalies, leading to embryonic death after 12.5 days of gestation [25]. However, its role as a maternal factor in early embryonic development remains poorly understood. In this study, we demonstrate that oocyte-specific knockout of IRE1α results in complete female infertility, with embryos arresting at the one-cell stage, underscoring the indispensable role of maternal IRE1α in early embryogenesis. Notably, ER stress levels did not increase from the GV stage to the one-cell stage, and deletion of IRE1α did not activate other UPR pathways, such as PERK and ATF6. Furthermore, mitochondrial function remained intact in IRE1α-GKO zygotes, despite prior reports implicating IRE1α in ER-to-mitochondria ion transport [29]. These findings suggest that the developmental arrest observed in IRE1α-deficient embryos is not due to an imbalance in ER homeostasis or mitochondrial dysfunction, indicating that IRE1α’s role in early embryonic development is independent of its traditional function in maintaining ER stress homeostasis.

As a maternal factor, the microinjection of *Ire1α* mRNA into *Ire1α*-GKO zygotes partially rescued the developmental arrest, whereas rescue attempts with IRE1α mutants (K907A and I642G) were unsuccessful, highlighting the necessity of IRE1α’s RNase activity for early embryonic development. While the IRE1α–XBP1 pathway regulates gene transcription [40] and has been implicated in embryonic development in medaka fish [31], we found that *Xbp1* mRNA levels and translation efficiency in mouse embryos remain low until the blastocyst stage. Overexpression of *Xbp1s* in *Ire1α*-GKO zygotes failed to rescue developmental defects, and inhibition of minor ZGA arrested embryos at the two-cell stage [41], indicating that the one-cell arrest in IRE1α-deficient embryos is not due to transcriptional inhibition or *Xbp1* splicing deficiency. Thus, IRE1α’s role in early embryogenesis appears to be independent of the classical IRE1α–*Xbp1* pathway.

In addition to its role in the IRE1α–XBP1 pathway, IRE1α directly cleaves RNA, a function implicated in diverse physiological contexts [42]. A pivotal event during early embryogenesis is the degradation of maternal mRNAs [1]. In IRE1α-deficient embryos, multiple maternal transcripts that are typically degraded during this period remained stable. Inhibition of IRE1α RNase activity similarly disrupted the degradation of these transcripts, indicating a direct role for IRE1α in maternal mRNA decay. Given that IRE1α target mRNAs have been identified in multiple myeloma cells and human embryonic kidney-derived cell lines [43, 44], we propose that IRE1α serves as a novel maternal factor regulating mRNA degradation in early embryos. Using IRE1α-Flag knock-in mice and the LACE-seq technique, we identified multiple maternal mRNAs directly bound by IRE1α in zygotes. And we further validated that IRE1α directly cleaved these maternal mRNAs *in vitro*. Further analysis revealed that these mRNAs contain CUGCAG-like motifs and form hairpin structures at IRE1α binding sites, and these sites could be cleaved *in vitro*.

Multiple factors regulate maternal mRNA degradation during the MZT, including CDK1-mediated MSY2 modulation [45], DCP1A/DCP2-mediated RNA cap structure alteration [46], and BTG-mediated recruitment of the CCR4–NOT complex [9]. After fertilization, major ZGA is involved in regulating maternal mRNA degradation beyond the two-cell stage [11, 47]. However, the mechanisms governing mRNA degradation from fertilization to ZGA, particularly at the one-cell stage, remain poorly characterized. Our findings reveal that IRE1α is specifically expressed at the one-cell and two-cell stages and directly degrades maternal mRNAs during this period. This discovery not only expands the understanding of IRE1α’s non-canonical functions independent of the UPR, but also provides new insights into maternal mRNA regulation in early embryogenesis.

Early embryonic development involves a series of coordinated events, including maternal mRNA degradation, histone modification, and ZGA. We found that aberrant maternal mRNA degradation was accompanied by disruptions in ZGA, histone modification, and DNA methylation, which may be secondary effects of impaired maternal mRNA decay caused by IRE1α deficiency.

In conclusion, our findings demonstrate that ERK1/2 regulates IRE1α translation during oocyte maturation. IRE1α directly degrades maternal mRNAs after fertilization, and its loss leads to abnormal accumulation of specific maternal transcripts, triggering defects in ZGA and histone modifications, ultimately resulting in embryonic developmental arrest. These results highlight IRE1α as a key regulator of maternal mRNA degradation and early embryogenesis, independent of its canonical UPR functions.

## Supplementary Material

gkaf520_Supplemental_Files

## Data Availability

All data are available in the main text or the supplementary material. The raw sequence data reported in this paper have been deposited in the Genome Sequence Archive in National Genomics Data Center, China National Center for Bioinformation/Beijing Institute of Genomics, and Chinese Academy of Sciences (GSA: CRA022770) that are publicly accessible at https://ngdc.cncb.ac.cn/gsa.
