# Supplementary material for: IRE1α RNase activity is critical for early embryo development by degrading maternal transcripts
Source: Nucleic Acids Res. 2025 Jun 18;53(11):gkaf520. doi: 10.1093/nar/gkaf520 (PMC12203788; doi:10.1093/nar/gkaf520)
Supplement: gkaf520_Supplemental_Files [file gkaf520_supplemental_files.zip › Supplementary+Material1.pdf]

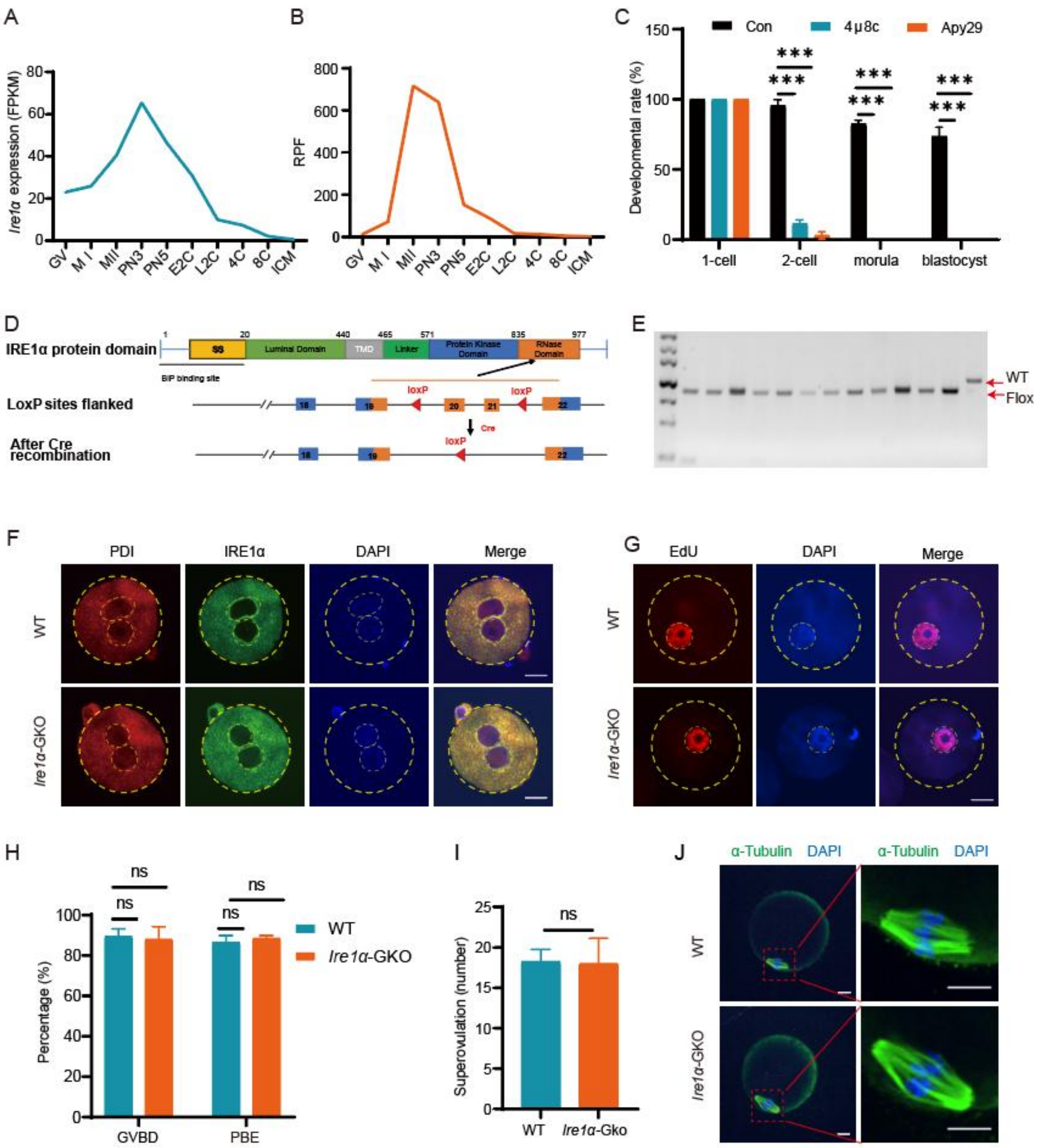

Figure S1: The oocytes of IRE1α-GKO female mice exhibit normal phenotype. (A) Line plots showing IRE1α mRNA levels during early embryo development from a previous publication. (B) Line plots showing IRE1α translation levels during early embryo development from a previous publication. RPF, ribosome-protected fragment. (C) Developmental rates of embryos treated with DMSO, 4μ8c and BI09. ****P* < 0.001 by two-tailed Student's test. (D) Schematic diagram showing the IRE1α conditional knockout strategy. (E) genotyping results (lower panel) showing the IRE1α-flox mice. (F) The immunofluorescence of PDI and IRE1α in zygotes derived from oviducts of WT and *Ire1α*-GKO mice. scar bar, 20 μm.(G) The EdU staining in zygotes derived from oviducts of WT and *Ire1α*-GKO mice. scar bar, 20 μm. (H) The

number of oocytes collected from oviducts of WT and *Ire1α*-GKO mice at 16 h after hCG injection. (I) Confocal microscopy results of MII oocytes collected from oviducts of WT and *Ire1α*-GKO mice at 16 h after hCG injection. Scale bar, 5μm. (J) Rates of GVBD and PB1 emission in oocytes derived from WT and *Ire1α*-GKO female mice.

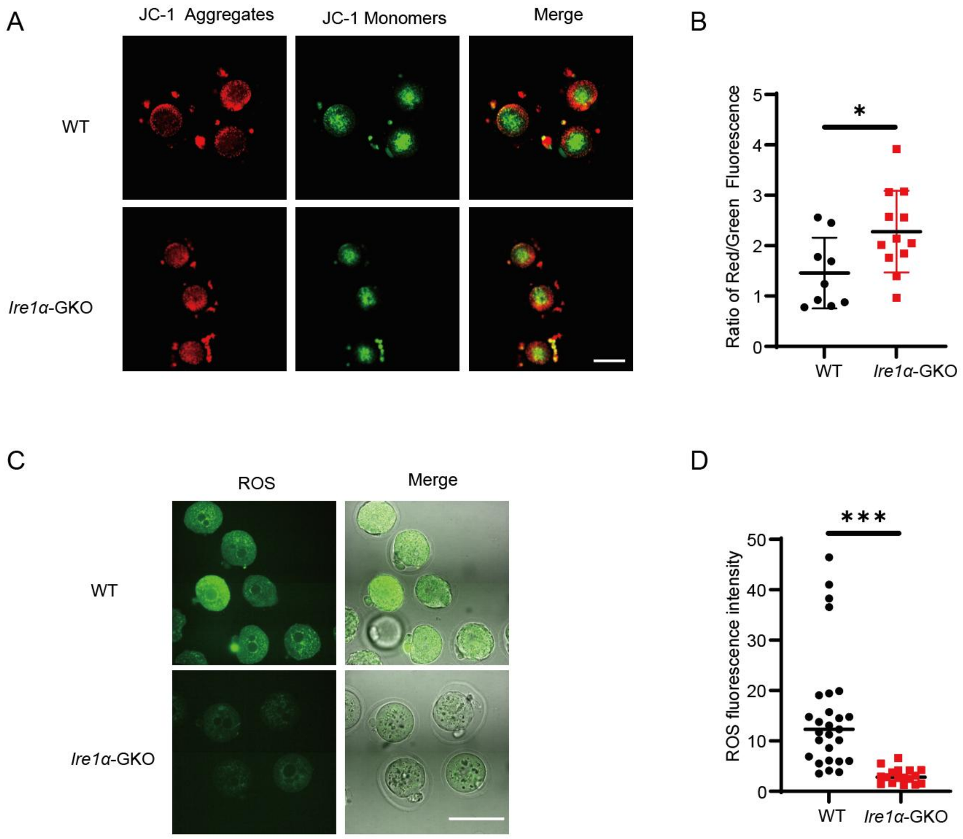

Figure S2: *Ire1α* deletion does not affect mitochondria function. (A) Images of JC-1 staining in WT and *Ire1α*-GKO zygotes. Scale bar, 100μm. (B) Relative fluorescence intensity statistics of JC-1 staining in WT and *Ire1α*-GKO zygotes, ****P* < 0.001, P-values were determined by a two-tailed unpaired t-test. (C) Images of ROS staining in WT and *Ire1α*-GKO zygotes. Scale bar, 100μm. (D) Relative fluorescence intensity statistics of ROS staining in WT and *Ire1α*-GKO zygotes, **P* < 0.05, P-values were determined by a two-tailed unpaired t-test.

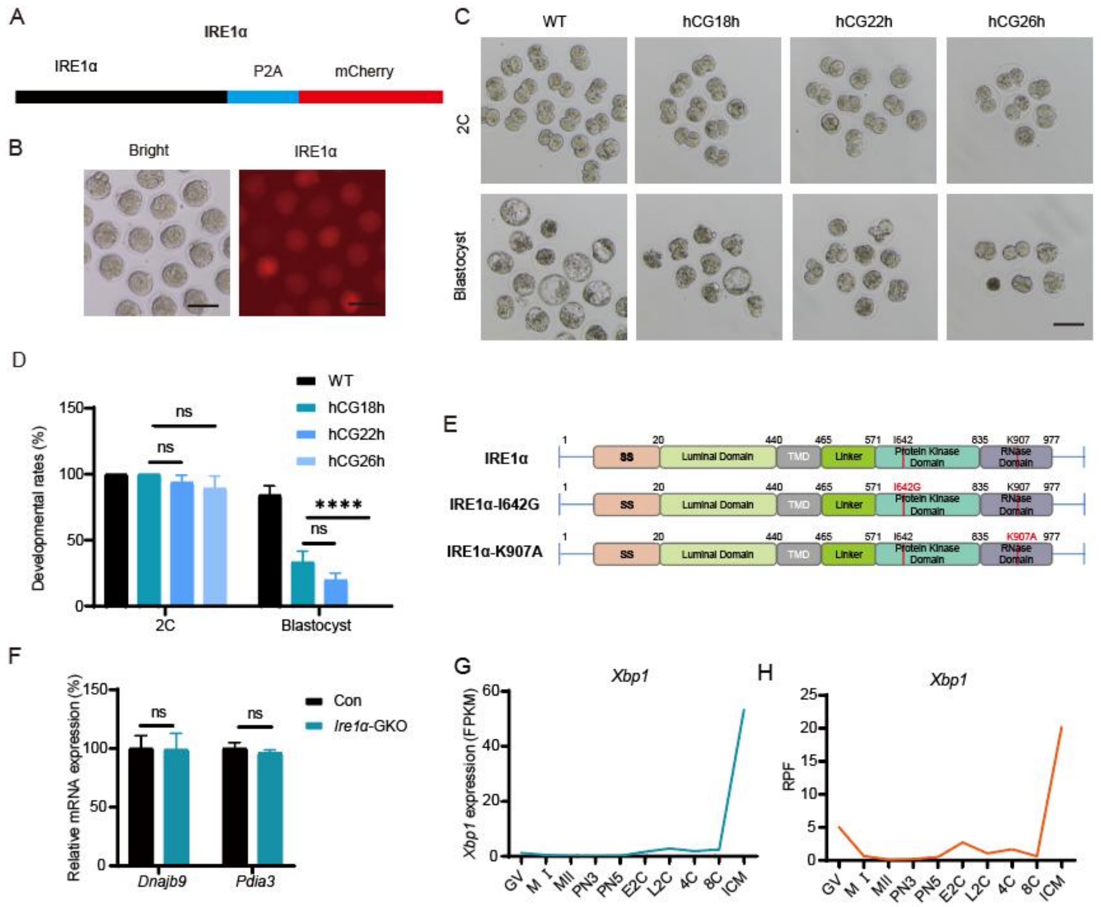

Figure S3: IRE1α RNase domain regulates early embryonic development through the RIDD pathway. (A) A diagram showing the protein structure encoding by *Ire1α* mRNA. (B) *Ire1α* mRNA were expressed in zygotes by microinjection. Scale bar, 100μm. (C) Developmental stages of IRE1α-GKO embryos injected with *Ire1α* mRNA at different time points. (D) Graph showing the embryonic developmental rate statistics of IRE1α-KO embryos injected with *Ire1α* mRNA at different time points. (E) A diagram showing the mutant site in major functioning domains of mouse IRE1α protein. (F) RT-PCR showing the relative mRNA expression of gene regulated by XBP1. (G) Line plots showing *Xbp1* mRNA levels during early embryo development from a previous publication. (H) Line plots showing XBP1 translation levels during early embryo development from a previous publication. RPF, ribosome-protected fragment.

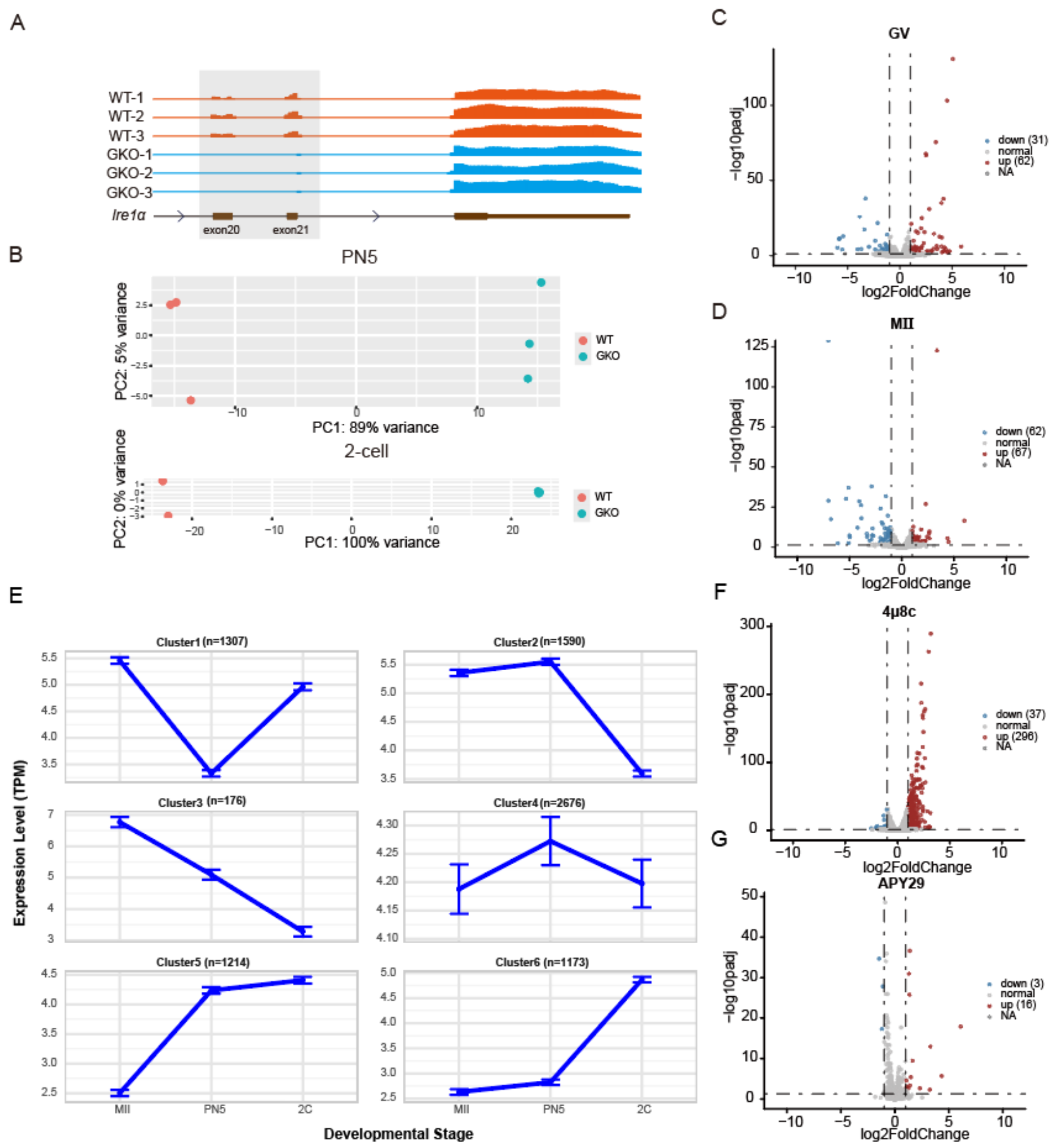

Figure S4: Transcriptomic changes in embryos following the loss of IRE1's RNase activity. (A) Single-cell RNA-seq results showing the complete deletion of exon 20 and exon 20 in *Ire1α*-GKO zygotes. Three independent scRNA-seq experiments are shown. (B) The PCA analysis of WT and *Ire1α*-GKO zygotes. (C), (D) Volcano plot showing transcriptome changes in *Ire1α* knock out GV and MII oocytes. (E) Expression pattern of mouse maternal transcripts at MII oocytes., PN5 zygotes, and late two-cell stages. (F, G) Volcano plot showing transcriptome changes in 4μ8c (F) and APY29 (G) treated PN5 oocytes.

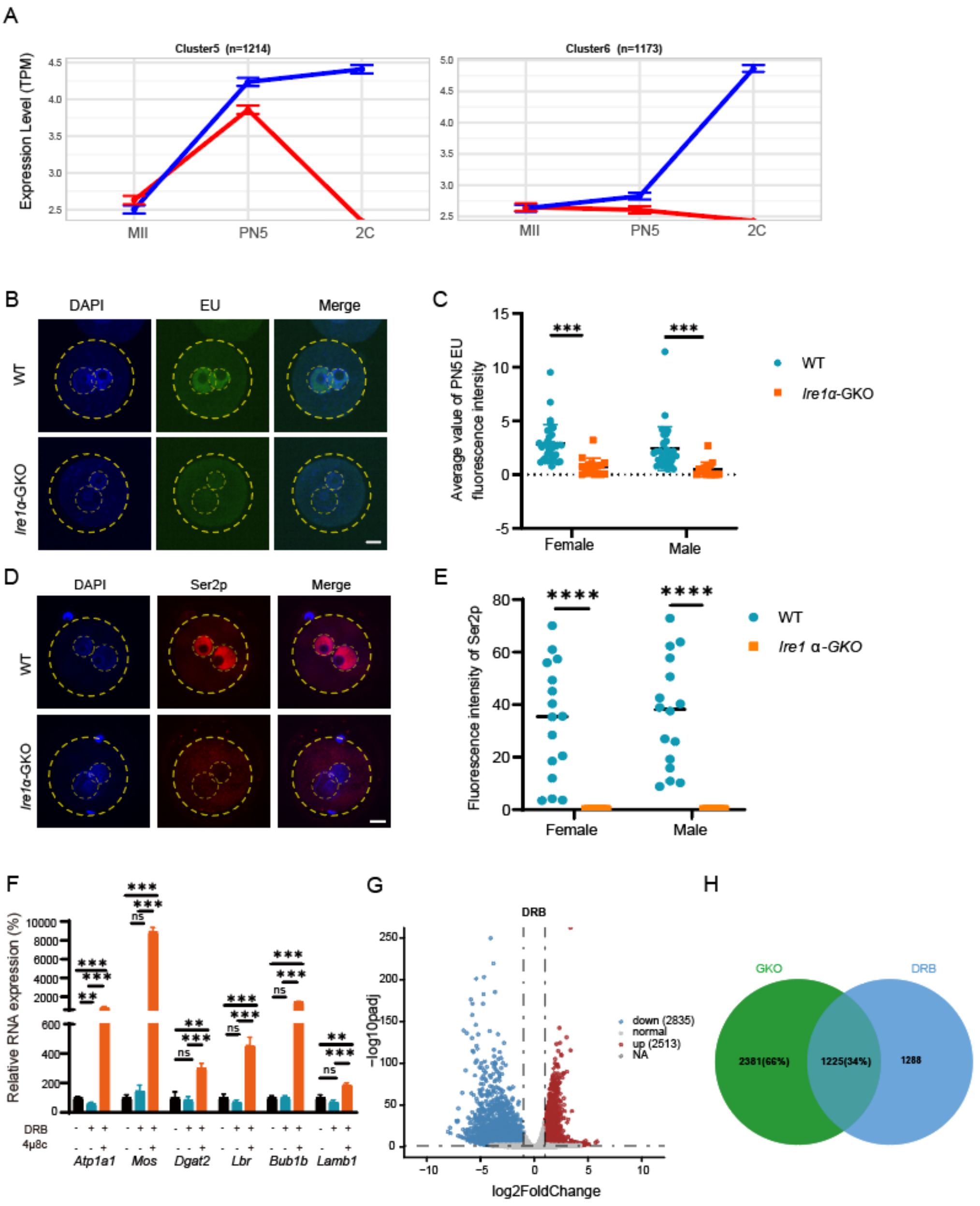

Figure S5: The abnormal maternal RNA degradation is not due to impaired transcription in *Ire1α*-GKO embryo. (A) The transcription pattern of minor and major ZGA in mouse embryos derived from WT and *Ire1α*-GKO females. (B) EU fluorescent staining of zygotes from WT and *Ire1α*-GKO females. Scale bar, 20μm. (C) Fluorescence intensity statistics for EU in WT and *Ire1α*-GKO zygotes. *** $P <$ 0.001 by two-tailed Student's t-tests. (D) Immunofluorescence staining of pol II Ser2 phosphorylation (Ser2p, red) and DNA (blue) in WT and WT and *Ire1α*-GKO zygotes. Scale bar, 20μm. (E) Fluorescence intensity statistics of Ser2p in WT and *Ire1α*-GKO

zygotes. ***P < 0.001 by two-tailed Student's t-tests. (F) RT-PCR showing the relative RNA expression transcripts in PN5 zygotes treated with DRB and 4μ8c. (G) Volcano plot showing transcriptome changes in 2-cell treated with DRB. (H) Venn diagrams showing the overlap of up regulated transcripts between DRB treated and *Ire1α*-GKO 2-cell.

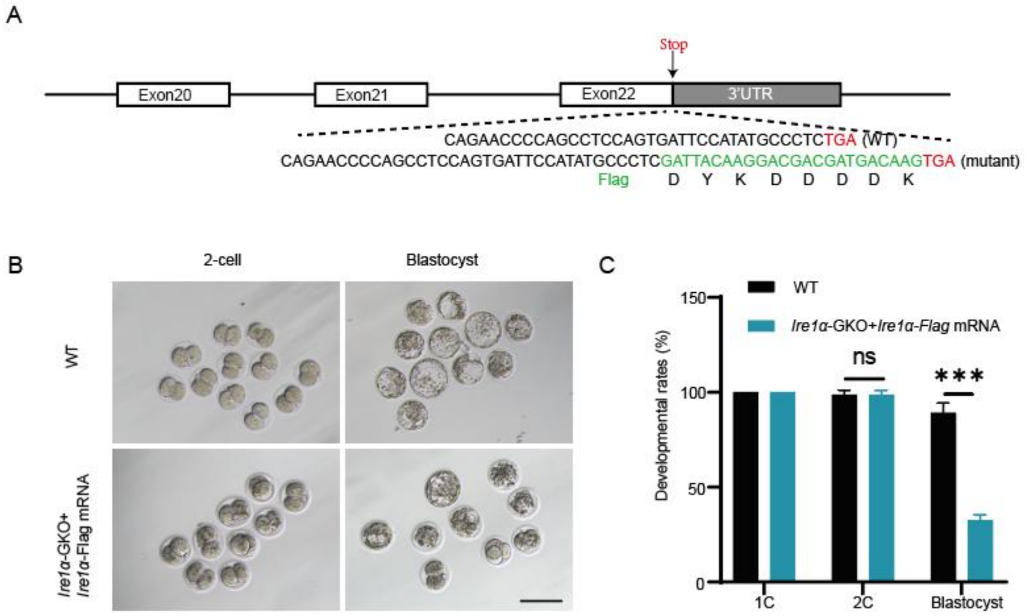

Figure S6: IRE1α-Flag mRNA rescues development arrest of embryos derived from *Ire1α*-GKO females. (A) Gene-targeting strategy for *Ire1α*-flag knock-in alleles. (B) Representative images of WT and *Ire1α*-GKO+*Ire1α*-flag mRNA (microinjection of *Ire1α*-flag mRNA mRNA in *Ire1α*-GKO zygotes) embryos. Scale bar, 100μm. (C) Statistics of the proportion of WT and *Ire1α*-GKO+*Ire1α*-flag mRNA embryos development when WT embryos reached the corresponding stages. ***$P < 0.001$, as assessed by two-tailed Student's t test.

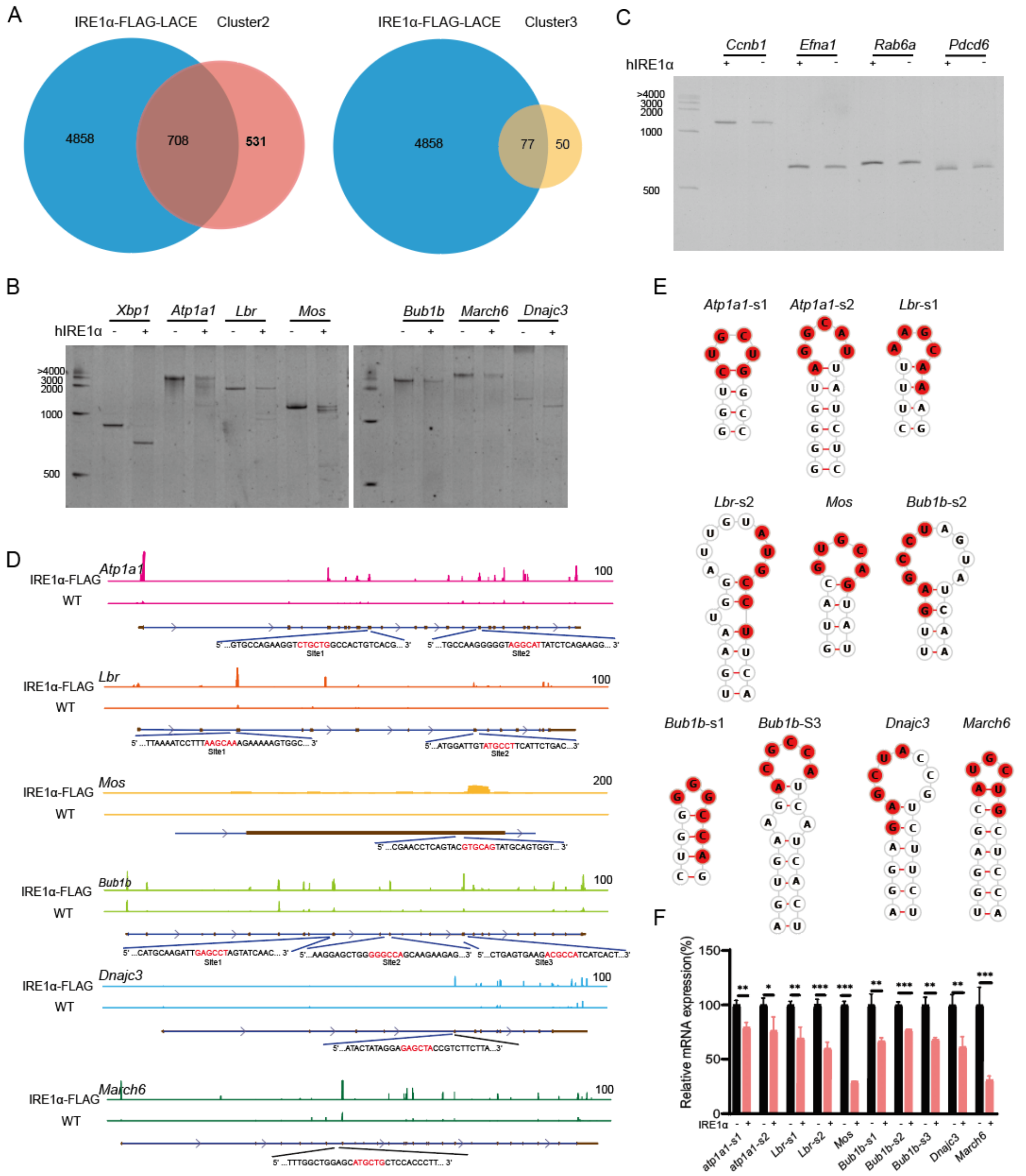

Figure S7: IRE1α RNase domain regulates maternal RNA decay through the RIDD pathway. (A) The overlapping of IRE1α-target genes (IRE1α-FLAG-LACE) with the maternal mRNA transcripts stabilized in IRE1α-GKO embryos (cluster2 and cluster3). (B) Urea-pages showing the IRE1α target mRNA fragments after treated with recombinant hIRE1α protein in vitro. (C) Urea-pages showing the non-IRE1α target mRNA fragments after treated with recombinant hIRE1α protein in vitro. (D) Genome browser snapshot showing the read coverage of the gene locus in IREα-Flag LACE-seq data. The Nucleotide sequences indicating all potential cleavage sites in selective RNA. Red bases indicating motifs recognized by IREα. (E) Hairpin

structures formed by all potential cleavage sites. Red bases indicating motifs recognized by IRE1. (F) RT-PCR showing the potential cleavage sites were cleaved by IREα in vitro. $^{*}P < 0.05$, $^{**}P < 0.01$ and $^{***}P < 0.001$ as assed by two-tailed Student's *t*-tests.

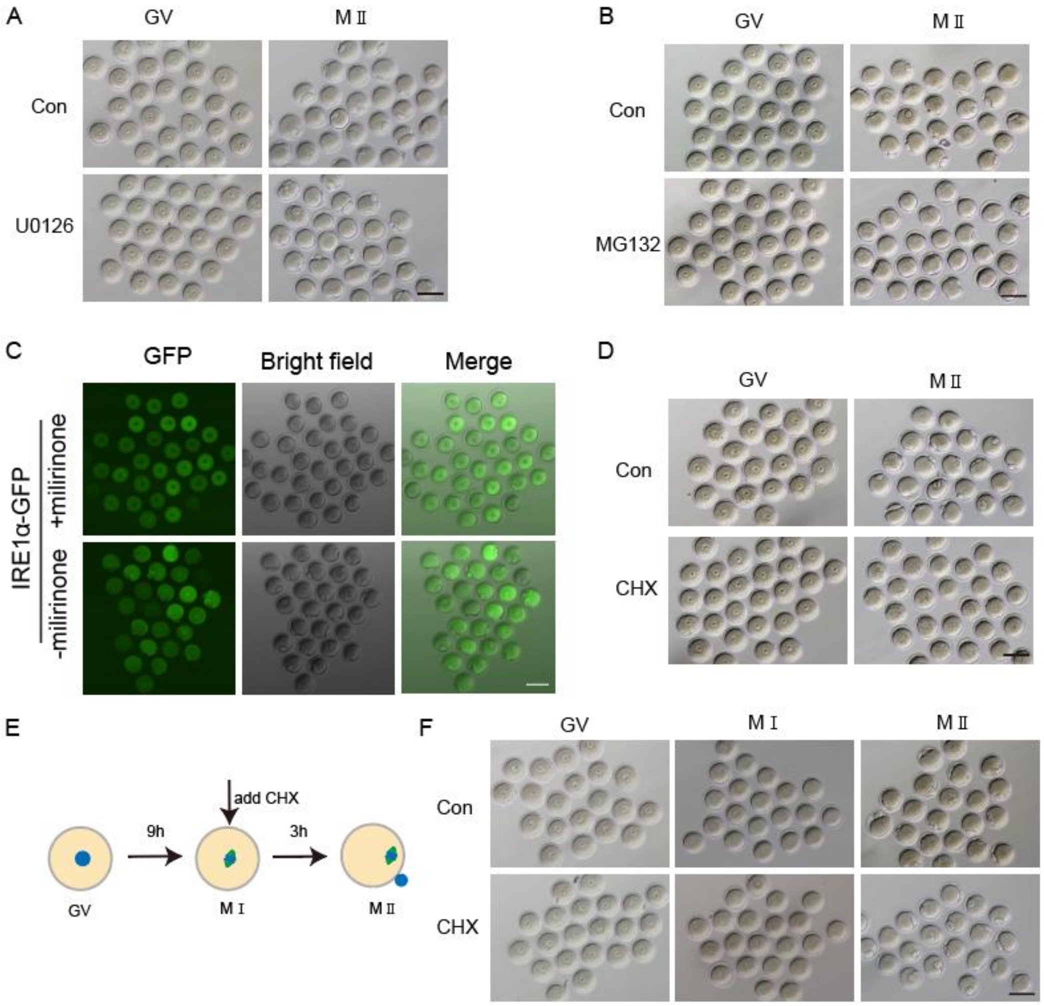

Figure S8: The effects of different inhibitors on the progression of oocyte meiosis. (A) U0126 treatment did not impair oocyte maturation to MII. (B) MG132 treatment impaired oocyte maturation to the MII stage. (C) Epifluorescence result showing that injected mRNAs encoded for IRE1α-GFP were stably expressed in GV oocytes and MII oocytes. (D) CHX treatment impaired oocyte maturation to the MII stage. (E) Schematic diagram of CHX treatment timing in oocyte maturation in vitro. (F) The developmental images of oocytes treated with CHX at the MI stage. Scale bar: 100μm.

Table S1. Primer sequences used for genotyping in this study

Gdf9-cre-F	AGGCATGCTTGAGGTCTGAT
Gdf9-cre-R	GAGATGTCCTTCACTCTGATTC
Ire1α-F	CCAGTGCTCTTGAAAAGAGG
Ire1α-R	CCCTGCCAGGATGGTCATGG
Ire1α-flag-F	TCCAACACACGGCCTTCATA
Ire1α-flag-R	CATGAGGCAACACGGGCTAT
Xbp1-F	ACACGCTTGGGAATGGACAC
Xbp1-R	CCATGGGAAGATGTTCTGGG

Table S2. Sequences used for IRE1α-Flag knock in mouse in this study

sgRNA1	GTGGCCCCAATAATGACCAT
sgRNA2	CGGGCCCATGGTCATTATTG
DNA donor	GCACGAGCCCACAGAACCCCAGCCTCCAGTGATTCCATATGCCCT CGATTACAAGGACGACGATGACAAGTGGCCCGATCTCTGCAGTC ATAGTTTGTTGCCTCTGGGATTAGCAGGAA

Table S3. Primer sequences used for qRT-PCR in this study

Gapdh-qF	AGGTCGGTGTGAACGGATTTG
Gapdh-qR	TGTAGACCATGTAGTTGAGGTCA
Xbp1-qF	TGGACTCTGACACTGTTGCCTC
Xbp1-qR	TAGACCTCTGGGAGTTCCTCCA
Mos-qF	AGTGGTTGCCTACAATCTGCGC
Mos-qR	GAGGTCCCTTTGGAGCAGTTCT
Lbr-qF	TGCTCCGTCCTTGGCAATGATC
Lbr-q*R*	AAAGCCGTCGTGCATGATGTCC
Bub1b-qF	GTCCACAGGTTCTCAATGCCCA
Bub1b-qR	TGATGGCGTCTTCACTCAGAGG
Atp1a1-qF	CCGTGGATAACCTCTGCTTCGT
Atp1a1-qR	CGCTGTGATTGGATGGTCTCCT
Dgat2-qF	CTGTGCTCTACTTCACCTGGCT
Dgat2-qR	CTGTGCTCTACTTCACCTGGCT
Lamb1-qF	GAACTACACGGTGAGGTTGGAG
Lamb1-qR	GAACTACACGGTGAGGTTGGAG
Ccnb1-qF	AGAGGTGGAACTTGCTGAGCCT
Ccnb1-qR	GCACATCCAGATGTTTCCATCGG
Atp1a1-splice-qF-1	CATCCACCTCATCACCGGG
Atp1a1-splice-qR-1	GTGAGTGTCAGACAGACCGT
Atp1a1-splice-qF-2	CATCCAATCACAGCGAAAGCC
Atp1a1-splice-qR-2	GGTTCACCTGGTTCACTGGA
Lbr-splice-qF-1	AGCCACGACAACAAATCCCA
Lbr-splice-qR-1	TCGAGAGGGAGAGCTGGAAA
Lbr-splice-qF-2	GGCGTTTACCTCCTCTGGTT
Lbr-splice-qR-2	AGGTAGCACAGCTCAACACC
Mos-splice-qF	AAAGGAGAGATTGCCACGCC
Mos-splice-qR	TGCAGTGTCTTTCCAGTCAGG
Bub1b-splice-qF-1	ACAATCCAGCCTCTGTGACG
Bub1b-splice-qR-1	TCTGCAAGGGGTCTCCTTCT
Bub1b-splice-qF-2	AAGAACGAAGGGAAGCCGAG
Bub1b-splice-qR-2	TGGGTCCTCTGTAGGCATCT
Bub1b-splice-qF-3	ACAGAAGTGGGCACCACAAA
Bub1b-splice-qR-3	GGTTGGGACAGAGAGTGACG
Dnajc3-splice-qF	AAATTACTCGCAGCCGGACA
Dnajc3-splice-qR	CCTCTCTGTAGTCTTGCGGC
March6-splice-qF	GGCTGTTTTGTGGTGACGTG
March6-splice-qR	TTCTCACCTGCTGGGTTAGC

Table S4. Primer sequences used for vector construct in this study

Ire1α-FL-F	TCACTATAGGGAGACCCAATGCCGGCCCGGTGGCT
Ire1α-FL-R	AGGCCCGGGGTTTTCTTCAACATCTCCTGCTTGCTTTAACAGAGAGAAG TTCGTGGCTCCGCTTCCGAGGGCATATGGAATCAC
mcherry-F	GGAAGCGGAGCCACGAACTTCTCTCTGTTAAAGCAAGCAGGAGATGTT GAAGAAAACCCCGGGCCTATGGTGAGCAAGGGCGAGGA
mcherry-R	GCGGCCGTTACTAGTGGATCTTACTTGTACAGCTCGTCCA
Ire1α-GFP-F	GCTGTACAAGGGTACCGAGCTCGGATCCATGCCGGCCCGGTGGCT
Ire1α-GFP-R	TCCTCCTCCAGATCCTCCAGATCCGAGGGCATATGGAATCAC
GFP-F	GGATCTGGAGGATCTGGAGGAGGAATGGTGAGCAAGGGCGAG
GFP-R	GCGGCCGTTACTAGTGGATCTCACTTGTACAGCTCGTCCAT
Ire1α-I642G-F	GGCGAGCTGTGTGCAGCCACCCTACAAGAG
Ire1α-I642G-R	AGCAATGTACTGGAACTGCCGGTCCTTCTC
Ire1α-K907A-F	AAACACCACTACCGGGAGCTCCCCGTGGA
Ire1α-K907A-R	AGCGTTTCTCATGGCTCGGAGGAGGTCTCTCAC

Table S5. Sequence parameter for LACE-seq adapter trimming

-a NNNNAGATCGGAAGAGCAC
-a NNNNAGATCGGAAGA
-a GGAAGAGCACACGTC
-a AGCACACGTCTGAAC
-a ACGTCTGAACTCCAG
-a TGAACTCCAGTCACC
-a TCCAGTCACCATTGC
-a TCACCATTGCTTATC
-a ATTGCTTATCTCGTA
-a TTATCTCGTATGCCG
-a TCGTATGCCGTCTTC
-a TGCCGTCTTCTGCTTG
-a G{"{30}"} -a A{"{20}"} -a T{"{20}"}
-G NNNNCAATCGNNNNNNNNNTTCAGACGTGTGCTCTTCCGATCT
-A NNNNAGATCGGAAGAGCGT
-A NNNNAGATCGGAAGA
-A GGAAGAGCGTCGTGT
-A AGCGTCGTGTAGGGA
-A CGTGTAGGGAAAGAG
-A AGGGAAAGAGTGTAG
-A AAGAGTGTAGATCTC
-A TGTAGATCTCGGTGG
-A ATCTCGGTGGTCGCC
-A GGTGGTCGCCGTATCATT
-A G{"{30}"} -A A{"{20}"} -A T{"{20}"}
