# Supplementary figures and images for: IRE1α RNase activity is critical for early embryo development by degrading maternal transcripts
Source: Nucleic Acids Res. 2025 Jun 18;53(11):gkaf520. doi: 10.1093/nar/gkaf520 (PMC12203788; doi:10.1093/nar/gkaf520)

Figure 1B

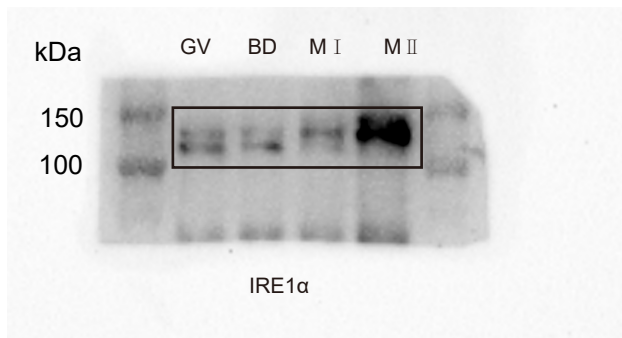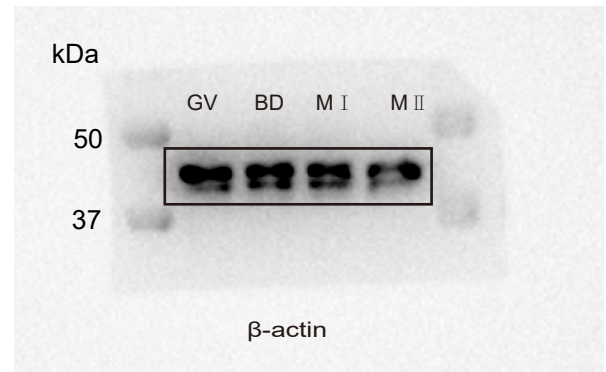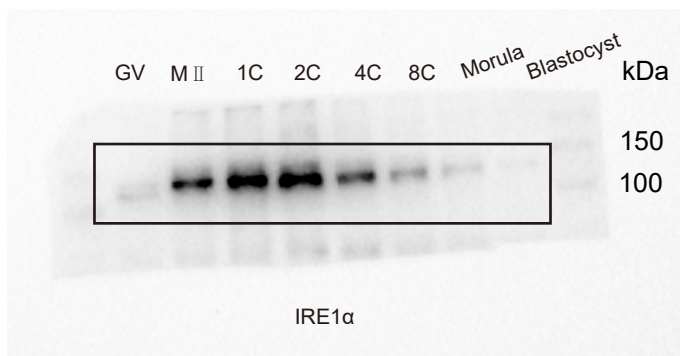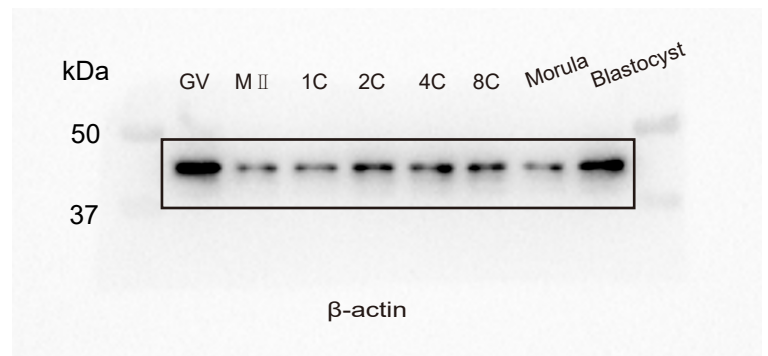

Figure 1D

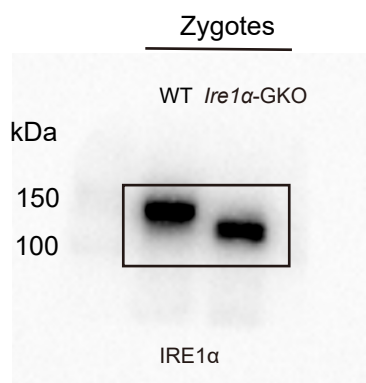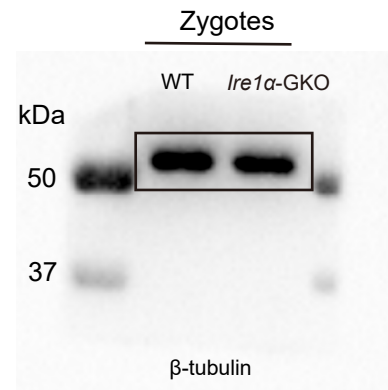

Figure 2A

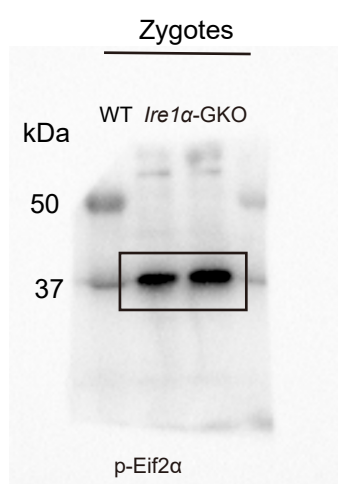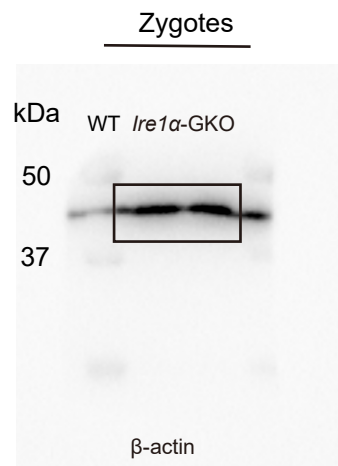

Figure 7A

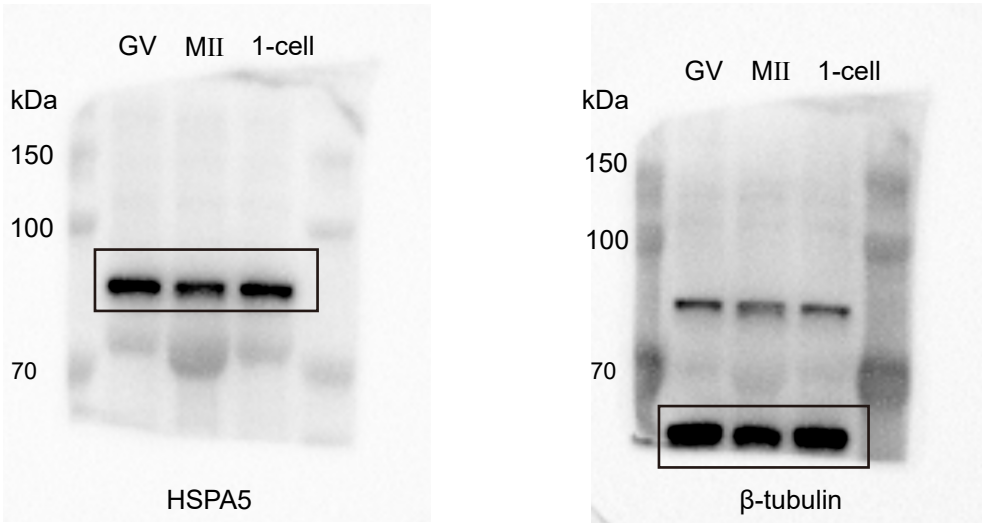

Figure 7B

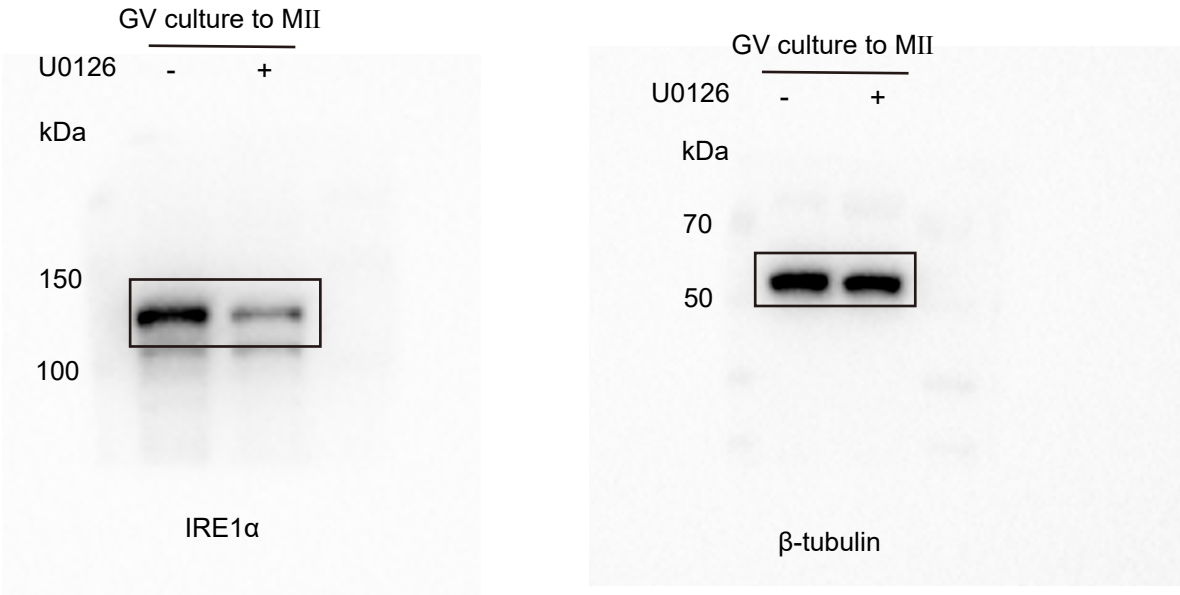

Figure 7C

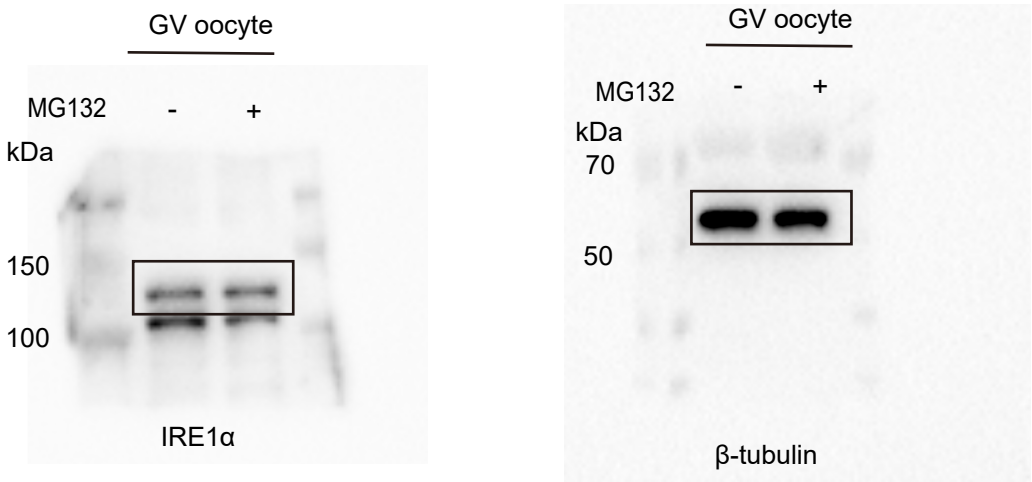

Figure 7D

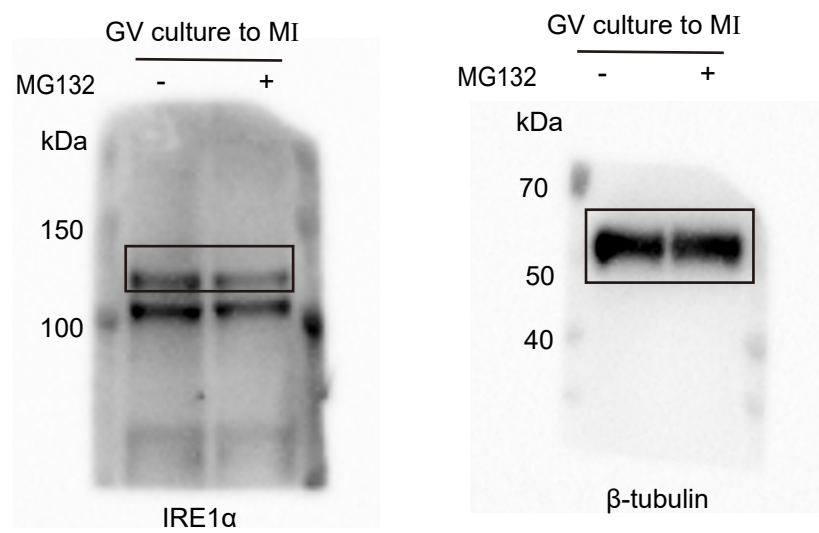

Figure 7E

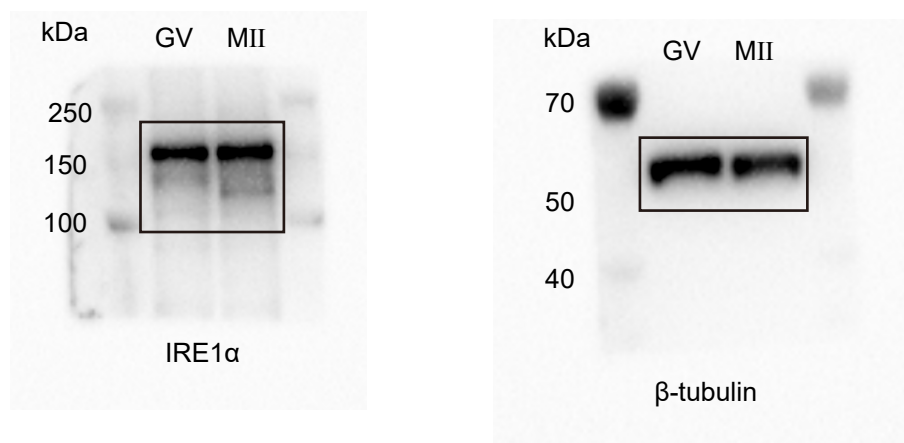

Figure 7F

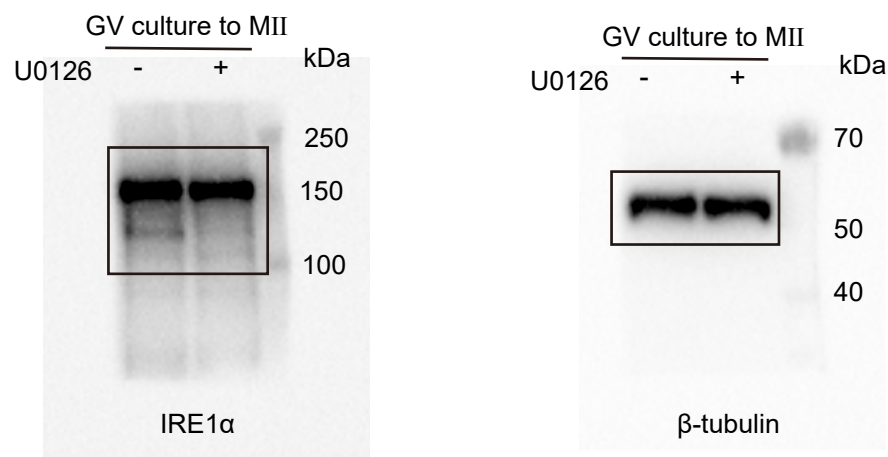

Figure 7G

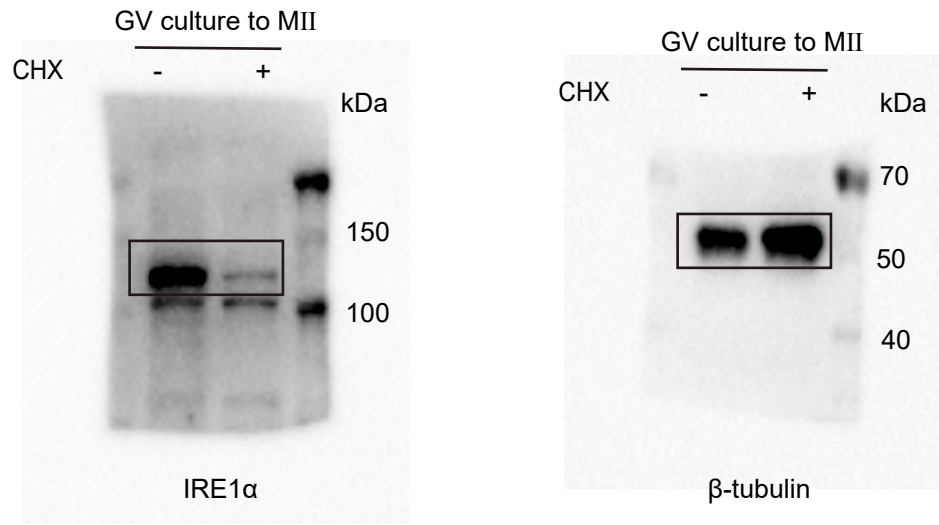

Figure 7H

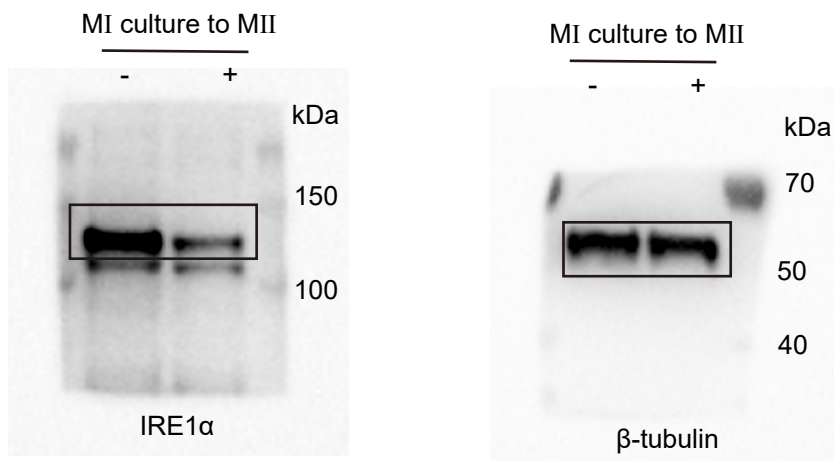

Supplement: gkaf520_Supplemental_Files [file gkaf520_supplemental_files.zip › Supplementary+Material2.pdf]
